# A Functional Phylogenomic View of the Seed Plants

**DOI:** 10.1371/journal.pgen.1002411

**Published:** 2011-12-15

**Authors:** Ernest K. Lee, Angelica Cibrian-Jaramillo, Sergios-Orestis Kolokotronis, Manpreet S. Katari, Alexandros Stamatakis, Michael Ott, Joanna C. Chiu, Damon P. Little, Dennis Wm. Stevenson, W. Richard McCombie, Robert A. Martienssen, Gloria Coruzzi, Rob DeSalle

**Affiliations:** 1 Sackler Institute for Comparative Genomics, American Museum of Natural History, New York, New York, United States of America; 2 Cullman Program in Molecular Systematics, The New York Botanical Garden, Bronx, New York, United States of America; 3 Center for Genomics and Systems Biology, Department of Biology, New York University, New York, New York, United States of America; 4 Department of Computer Science, Technische Universität München, Munich, Germany; 5 Department of Entomology, University of California Davis, Davis, California, United States of America; 6 Cold Spring Harbor Laboratory, Cold Spring Harbor, New York, United States of America; University of Arizona, United States of America

## Abstract

A novel result of the current research is the development and implementation of a unique functional phylogenomic approach that explores the genomic origins of seed plant diversification. We first use 22,833 sets of orthologs from the nuclear genomes of 101 genera across land plants to reconstruct their phylogenetic relationships. One of the more salient results is the resolution of some enigmatic relationships in seed plant phylogeny, such as the placement of Gnetales as sister to the rest of the gymnosperms. In using this novel phylogenomic approach, we were also able to identify overrepresented functional gene ontology categories in genes that provide positive branch support for major nodes prompting new hypotheses for genes associated with the diversification of angiosperms. For example, RNA interference (RNAi) has played a significant role in the divergence of monocots from other angiosperms, which has experimental support in *Arabidopsis* and rice. This analysis also implied that the second largest subunit of RNA polymerase IV and V (NRPD2) played a prominent role in the divergence of gymnosperms. This hypothesis is supported by the lack of 24nt siRNA in conifers, the maternal control of small RNA in the seeds of flowering plants, and the emergence of double fertilization in angiosperms. Our approach takes advantage of genomic data to define orthologs, reconstruct relationships, and narrow down candidate genes involved in plant evolution within a phylogenomic view of species' diversification.

## Author Summary

Understanding the genetic and genomic basis of plant diversification has been a major goal of evolutionary biologists since Darwin first pondered his “abominable mystery,” the rapid diversification of the angiosperms in the fossil record. We develop and deploy a functional phylogenomic approach that helps identify genes and biological processes putatively involved in species diversification. We assembled a matrix of 22,833 orthologs from 150 species to reconstruct seed plant phylogenetic relationships and to identify gene sets with a unique evolutionary signal. Our analysis of overrepresented biological processes in these sets narrowed down possible genetic mechanisms underlying plant adaptation and diversification. The phylogenetic relationships we uncovered support the hypothesis that gnetophytes are closely related to the rest of the gymnosperms at the base of the living seed plants. We also found that genes involved in post-transcriptional silencing via RNA interference (RNAi)—increasingly important in understanding plant evolution—are significantly represented early in angiosperm and gymnosperm divergence, with an apparent loss of specific classes of small interfering RNAs (siRNA) in gymnosperms. Our functional phylogenomic approach can be applied to any taxa with available sequences to enhance our knowledge of the evolutionary processes underlying biodiversity in general.

## Introduction

Attempts to clearly resolve the relationships among major seed plant groups using nuclear gene sequences have been hampered by the small number of completely sequenced genomes, the scarcity of ESTs for certain plant groups, and the lack of automated tools that can assemble and analyze large phylogenomic data sets. Existing phylogenetic hypotheses from molecular data, are often disputed due to the small sample of genes and/or taxa used in the analyses, regardless of the degree of support. Various conflicting topologies for the five basic seed plant groups have been obtained over time [1], [2]. Plant molecular phylogenetics has long relied on plastid genomes and only a few nuclear markers to infer relationships [1], [3]–[9]. Recently, progress has been made in generating plastid genome-based plant phylogenies [3], [10]–[16], but nuclear genome-scale analyses of plants have only recently started appearing in the literature [17]–[19]. The incorporation of nuclear phylogenomic information in plant phylogenetics would accomplish two important goals. First, the phylogenetic patterns discovered using nuclear genomic information could be used to corroborate the many well-supported plastid relationships, and to shed light on those relationships that are still at odds. Second, nuclear phylogenomic information can be used to derive new hypotheses for the function of plant genes that are relevant to major divergence events in plant evolution. In this study, we use phylogenetic information (emergent measures of phylogenetic support [20]) as the platform to identify candidate genes that may have played a role in plant adaptation. We first identify sets of orthologs from genomic sequences using a phylogenetic context [21]. We next use these orthologs to construct a total-evidence phylogeny and examine the distribution of their support metrics per node [20]. We then assess the statistical significance of Gene Ontology (GO) categories for gene lists that provide positive phylogenetic support to a node with functional processes of interest (e.g. seed development) [22]. The main premise of this approach is that genes (partitions) that are in agreement or in conflict with the overall evolutionary history of a particular node in a phylogeny, can be detected and used to derive hypotheses for the genes and biological processes potentially responsible for some of the more interesting organismal differences among the taxa in a phylogenetic analysis. We thus employ a phylogenomic approach to postulate hypotheses of gene function distributions and evolutionary mechanisms. These hypotheses can be validated experimentally in follow-up studies, focusing the effort of finding candidate genes and planning downstream experiments on those candidates based on a phylogenomic context. This functional phylogenomic approach is fundamentally different than classical phylogenetic analysis methods and also from current functional genomic methods that mine genomic information without incorporating a phylogenetic context in their search for both orthologs and candidate genes of functional importance. The present study is also a step toward generating an automated phylogenomic method for the entire nuclear component of plant genomes. Here, we use this approach to begin exploring and deriving hypotheses for the evolutionary mechanisms that underlie plant adaptation and diversification, as exemplified by the explosion of biodiversity within the seed plants, underlying Darwin's abominable mystery on the sudden appearance and rapid diversification of flowering seed plants, but also on the persistence of the gymnosperms over evolutionary time.

## Results

### Inferred seed plant phylogeny

**Figure 1:**
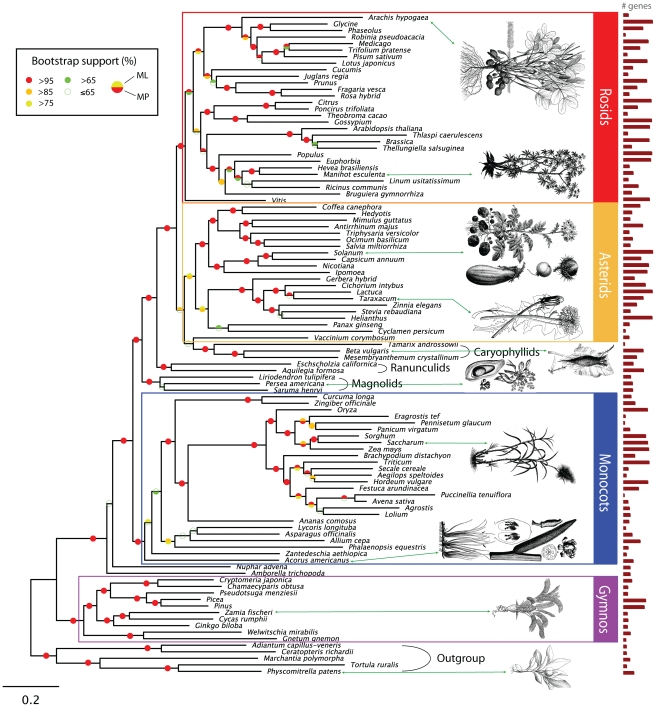
Maximum Likelihood Phylogram of the Genus-Only Alignment of 101 Taxa with at Least 30% Representation per Partition (ML-30) Using the GTR Substitution Matrix and the CAT Model of Among-Site Rate Heterogeneity. Taxon with genus-only label represents multiple species of the same genus. ML and MP bootstrap support percentages are color-coded. ML node values indicate the percentage of rapid bootstrap pseudoreplicates containing the nodes of the best ML tree. MP values correspond to the bootstrap proportions on the 50% majority-rule consensus tree. The red bars represent the relative number of genes per species represented in this matrix. The most-represented species in the full matrix (with respect to number of genes) is *Glycine max* (10,071 genes), and the least-represented species is *Puccinellia tenuiflora* (173 genes; see Table S1). The median number of gene partitions in which a taxon is represented is 2,071. This ML-30 tree has 101 taxa (genera), derived from 2,970 gene partitions and 1,660,883 characters. Outgroups include the ferns, *Adiantum* and *Ceratopteris*; the mosses *Physcomitrella* and *Tortula*; and the liverwort, *Marchantia*. The estimated GTR substitution matrix is provided in Table S5.

We used OrthologID (OID) [21] – a program for automated, parsimony tree-based orthology determination, to identify 22,833 sets of orthologs from 150 plant species (see Materials and Methods for a description of the extended OID pipeline). These plant species, belonging to 101 different genera, represent a broad taxonomic range of angiosperms and extant gymnosperms. To reduce the size of the dataset for maximum likelihood (ML) analysis, and to remove partitions with the most missing data, we also constructed a matrix by only including genes with at least 30% representation across all genera. In this >30%-matrix, multiple taxa belonging to the same genus are collapsed into a single taxon. The average number of genera represented in each gene partition is 41 (40.6%) in this matrix. The cumulative distribution of gene partitions by taxon representation is shown in Figure S2. We performed maximum parsimony (MP) analysis on both matrices (MP-full and MP-30), as well as maximum likelihood analysis on the >30%-matrix (ML-30) (see Text S1). Figure 1 shows the phylogenetic tree generated from the ML-30 analysis.

The basic topologies for all three trees of the seed plants (MP-full, Figure S3; MP-30, Figure S4; and ML-30) are essentially identical. Node support based on bootstrap methods yielded a robust inferred phylogenetic tree overall. All three of our analyses (MP-full, MP-30, and ML-30) corroborate the same monophyletic groups of seed plants, as revealed in all previous morphological analyses and most molecular analyses, namely the seed plants, the cycads, the conifers, the gnetophytes, and the angiosperms. Moreover, all of our analyses support the gymnosperms as a monophyletic group (bootstrap  = 100%). This is congruent with all comparable molecular data sets to date [1], [5], [11], [23], and in contrast to most morphological analyses, which retrieve gymnosperms as paraphyletic [7], [24], [25]. The differences between molecular-based topologies of the gymnosperms mainly involve the placement of the gnetophytes, that is with the gnetophytes as sister to the conifers [5], nested in conifers [1], [11], [13], [16], as sister to all other gymnosperms [23], or sister to all other seed plants [13], [26]. The position of the gnetophytes among seed plants, is indeed one of the most interesting unresolved issues in plant systematics, as reviewed in [2]. Our inferred genome-wide phylogeny (bootstrap  = 100%) supports the gnetophytes as basal extant gymnosperms. This finding supports earlier hypotheses retrieved from individual gene trees such as *rpo*C1 and *rbc*L, as well as the non-coding regions of the inverted repeat representing the plastome [27]–[29], and from phytochrome genes [23], [30], *AGAMOUS*-like genes [31], [32], and *FLORICAULA*/*LEAFY* [33] representing the nuclear genome.

The topology of this phylogenomic view of the seed plants with a pectinate (i.e. maximally asymmetric, e.g. [34]) series of angiosperms, Gnetales, cycads + *Ginkgo*, and conifers has a impact on the interpretation of plant evolutionary changes, as characters are optimized in different ways. For example, in the previous view that cycads are sister to the rest of the gymnosperms, with ferns as sister to both flowering plants and gymnosperms, then the comparison is that angiosperms carpels are megasporophylls (seed-bearing sporophylls), and the angiosperm gynoecium is a simple strobilus (reproductive organ). By contrast, our phylogenomic view of the seed plants, Gnetales are sister to the rest of the gymnosperms with ferns as sister to all seed plants, and in this view each angiosperm carpel bearing ovules can be most parsimoniously interpreted as a simple strobilus. In this phylogenomic view, the angiosperm gynoecium would now be interpreted as a compound strobilus, with each carpel representing a bract enclosing an axillant ovule-bearing axis (Figure S6A). Another example of the impact of optimization is found with motile male gametes. Motile male gametes characterize all of the non-seed plant out-groups, and within seed plants are found only in cycads and *Ginkgo*. In our phylogenomic topology, motile male gametes would be independently and uniquely evolved (apomorphic) in cycads plus *Ginkgo*, and loss of motile male gametes in Gnetales and conifers would be ancestral in the gymnosperms (plesiomorphic). In contrast, if cycads were sister to the rest of the gymnosperms, the loss of motile male gametes would be convergent in conifers plus Gnetales and in the angiosperms, as an independently derived apomorphy. In this case, motile male gametes in cycads and *Ginkgo* would be a gymnosperm plesiomorphy (Figure S6B). Perhaps the most interesting aspect of these reversed character optimizations is that with the Gnetales sister to the rest of the gymnosperms as in our phylogenomic tree, the interpretation of character evolution is the same as in the Anthophyte hypothesis, where Gnetales is sister to angiosperms, and cycads are sister to all other seed plants. The optimization of motile male gametes of cycads and *Ginkgo* in our phylogenomic-based topology is thus equivalent to the optimization of characters required to explain the Gnetales as nested within the conifers. In this case, the reinsertion of the inverted repeat in the plastid genome of the Gnetales would be required after its loss in all of the conifers [11].

It is noteworthy that in this phylogenomic view of the seed plants, the basic topology of the angiosperm tree used by the Angiosperm Phylogeny Group (APG II, III) [35], [36] is supported with only minor changes (Figure S5). We retrieve the same topology of major groups on the pectinate backbone, starting with *Amborella* followed by *Nuphar*, magnoliids, ranunculids, caryophyllids, rosids, and asterids. The few discrepant nodes between our phylogenomic trees generated using different approaches (e.g. MP and ML), and between our tree and other major phylogenies, have low support, and are likely the consequence of ambiguous orthology statements due to missing data. For instance, *Vaccinium* is placed with low support (<65%) either sister to the caryophyllids in our ML-30 tree (Figure 1), or within the asterids (with caryophyllids as sister to both rosids and asterids) in the MP-30 tree (Figure S4), which is congruent with APG III (with caryophyllids as sister to asterids). Resolution of either hypothesis will probably depend on sampling the Cornales, of which core taxa have been placed within the asterids, and as sister to Ericales containing *Vaccinium* [35], [37]. Similarly, increased sampling of the alismatids and aroids will help confirm the position of *Acorus* within the angiosperms. Our findings of *Acorus* as sister to all other monocots, supports most analyses conducted with single and multiple gene trees [38]–[41], except for those that include *atp*A, which in some instances, place *Acorus* within the Alismatales aroids plus alismatids [42]–[44]. Within the rosids, the topology of MP-30 departs from that of APG III, by placing the Sapindales (*Citrus* and relatives) with the rosids I, versus rosids II in APG III and in our MP-full and ML-30 analyses. Our phylogenomic analysis identifies these controversial nodes and points to the taxa that need further sampling.

A controversial topology that is present and well supported (bootstrap  = 100%) in all our of phylogenomic trees is the placement of the monocots between *Nuphar* and magnoliids. The monophyly and placement of the magnoliids has been enigmatic for some time, although recent work has suggested some resolution. The position of the monocots with respect to magnoliids has also been controversial. The three principal competing hypotheses for the position of the magnoliids are as sister to the eudicot lineage [45]–[47], as sister to the monocots plus the eudicots [38], [39], and as sister to the monocots [41]–[43]. By contrast, our phylogenomic tree firmly places the magnoliids as sister to the eudicots, and the monocots as sister to the magnoliids plus the eudicots, both with high support (100%). In fact, bootstrap support percentages along the backbone (the pectinate portion) of the angiosperms, also show that overall, our phylogenomic analysis provides a robust topology for this important group, based on a broad sampling of genomic and EST data.

### Identifying candidate genes of functional significance within a phylogenomic framework

With the large number of genes in our phylogenomic matrix covering every Gene Ontology (GO) category for plants [48] (see Table S2), we are able to derive hypotheses for the functional evolution of genes in a phylogenomic context, by analyzing gene partitions that lend evidence of support at the major nodes on the tree [49]. Currently, one of the most widely used methods in testing for selection in phylogenomics, such as *dN*/*dS* [50], relies mostly on statistical methods of detection of natural selection. Partitioned phylogenetic support detects any positive or negative support that a gene or category of genes has on any given node on a phylogenetic tree. We propose that genes with positive partitioned support at nodes are a broader category of candidate genes for exploring function that would include genes evolving not only under positive Darwinian selection, but also under stochastic processes and even purifying selection. Once these candidate gene lists that provide positive branch support are derived, we can then examine them using positive selection scans to determine the functional categories of genes that are under selective pressure. Instead of requiring overwhelming statistical evidence of positive selection, our method identifies significant evolutionary trends by quantifying both phylogenetic congruence and incongruence, thus detecting potentially important genes that might be evolving neutrally or under negative selection. In this way, phylogenetic incongruence between a functional class of genes (e.g. RNA silencing genes) and the organismal phylogeny, would suggest that this given gene has experienced a unique evolutionary history relative to that of the organisms *per se*. Detection of such sequences is given by character information, meaning that no previous knowledge about the gene or gene function is required. Apart from being an unbiased approach, this method allows for the discovery of candidate genes with potential evolutionary and functional relevance, which can then be evaluated for evidence of selection using downstream validation and standard evolutionary tests [51], [52]. Here we provide a few examples of candidate genes for such validations.

For this analysis to be computationally feasible, and to exclude gene partitions with a large amount of missing taxa, while retaining as many partitions as possible for statistical analysis, we extracted a 9,787-gene matrix with >10% representation per partition and performed Partitioned Bremer Support (PBS) analysis [20]. This metric gives a relative measure of positive support on a gene-by-gene basis for each node. We identified 7,689 gene partitions with positive PBS values at one or more nodes in the simultaneous analysis tree. Of the 7,000+ genes, 4,803 of them have identifiable *Arabidopsis* orthologs, which were used to annotate the partitions with GO and MIPS [53] terms. To assign significance, we tested each node for overrepresented GO or MIPS categories within the list of gene partitions with positive PBS. As with any phylogenetic tree, missing genes as a result of incomplete EST coverage, and the proportion of matches to GO and MIPs categories, could shift PBS support. Importantly, the proportion of overrepresented genes with positive PBS is not just a numbers game, as it does not correlate with the number of genes per species in our matrix (e.g. Figure 1 shows *Glycine* and *Phaseolus* with a large EST set vs. *Cucumis* and *Juglans regia*, with a moderate EST set, but highly significant overrepresented genes with positive PBS). Furthermore, although the proportion of positive PBS varies within the phylogenomic matrix, it remains high even in nodes with lower numbers of genes and GO and MIPs terms (Figure S7).

**Figure 2:**
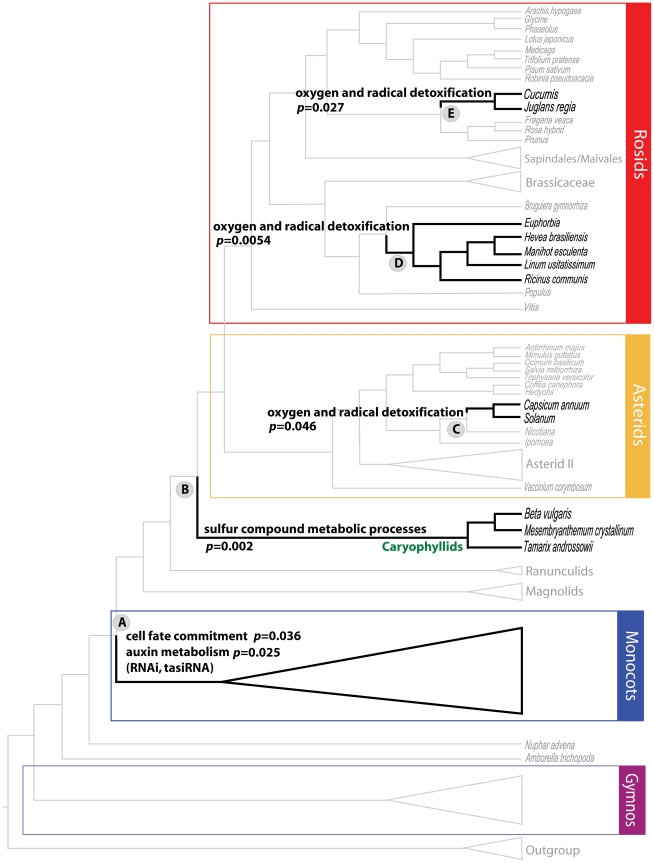
Select Overrepresented GO/MIPS Categories of Genes with Positive PBS at Major Nodes. There are statistically higher numbers of genes belonging to these GO/MIPS categories with positive support for the specific clades, implying that these genes may have special functional importance to the evolution of the corresponding clades. Only gene categories mentioned in the main text are shown. For a full list of overrepresented categories in each node see Table S3.

**Figure 3:**
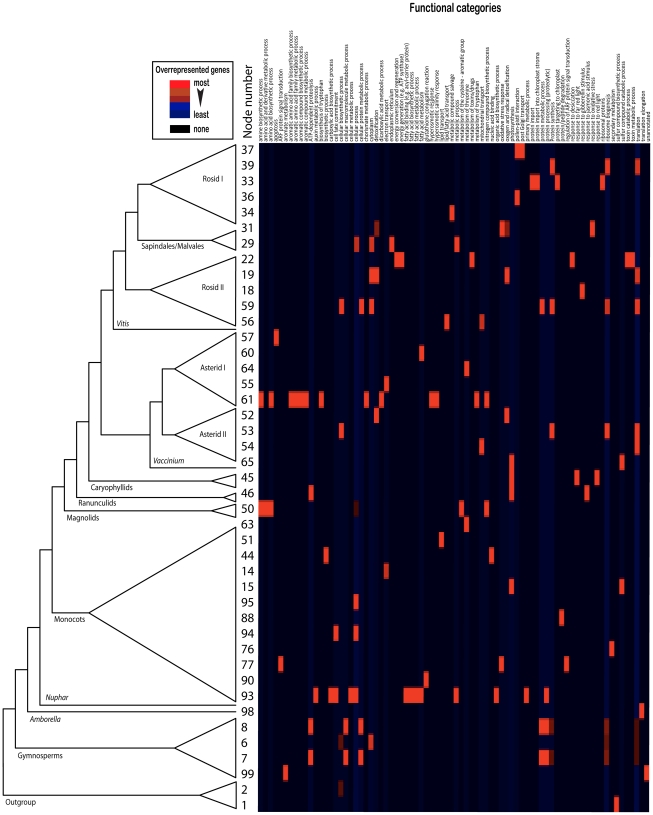
Distribution Map of Overrepresented GO Terms per Node. Each GO/MIPs category is shown in the upper axis. Color gradients show differences in proportions of these genes, with red being the category with the highest counts, light blue the least counts, and black with no match to any category. Overrepresentation is estimated on per-node basis. The reference tree is based on the MP-30 phylogenetic tree, and can be used to locate the relative position of a node represented by a heatmap row. The node numbering here corresponds to the node labels in Figure S7. Heat map constructed based on the *Arabidopsis* genome (source: http://noble.gs.washington.edu/prism – accessed on February 2009).

This analysis of genes providing positive branch support at key nodes in the phylogenomic tree identified 29 overrepresented GO/MIPS term-node pairs (*p*<0.01), 87 such pairs (*p*<0.05), and 138 (*p*<0.10) (see Table S3 for the complete list and Figure 2 and Figure 3 for the distribution map of overrepresented GO/MIPS terms). The significant overrepresentation of genes in these GO/MIPS terms, points to potential candidate genes involved in metabolic and developmental traits associated with the evolution of taxa within these clades. Note that overrepresentation in this context is unrelated to levels of gene expression, referring instead to the overrepresentation of proteins in each functional category among those with amino acid sequences contributing positive PBS to gene partitions.

An example of a node with an overrepresentation of genes involved in a metabolic process is Node 15, which includes the caryophyllids, members of which include the salt/drought-tolerant plants *Mesembryanthemum* and *Tamarix*. This node shows significant overrepresentation of genes involved in “sulfur compound catabolic processes” (*p* = 0.002), for which experimental data relates both genes in this GO term (MGL1 and GGT3), to the trait of drought stress in *Arabidopsis* [54]–[56]. The examination of GO overrepresentation in a phylogenomic framework, confirms expected patterns of well-characterized genes in some taxa, and also allows us to identify similar gene functions in atypical candidates. Genes involved in “oxygen and radical detoxification” were overrepresented in Node 52 (*p* = 0.046), Node 19 (*p* = 0.00054), and Node 31 (*p* = 0.027). Species like tomato (*Solanum lycopersicum*) [57] and pepper (*Capsicum annuum*) [58] in Node 52, *Euphorbia esula* and *E. tirucalli* [58], [59] in Node 19, and walnut (*Juglans regia*) [60] in Node 31, are well known sources of detoxifying and antioxidant compounds. The predominance of glutathione-related genes involved in detoxification [61] in those clades is not surprising and demonstrates our approach in principle. The overrepresentation of glutathione peroxidase genes in other taxa such as melon (*Cucumis melo*) and cucumber (*C. sativus*) in Node 19, is thus worth examining further.

Another example of this functional phylogenomic approach identified genes belonging to “cell fate commitment” (*p* = 0.04) and “auxin metabolism” (*p* = 0.025) as overrepresented GO terms among genes with positive PBS at Node A (Figure 2), that defines all monocots except *Acorus*. These include three genes that encode proteins involved in cell fate decision, *AGO1*, *KANADI* and *LACHESIS*. *LACHESIS* controls the cell fate within female gametophytes in *Arabidopsis*, where mutants have supernumerary egg cells and are semi-sterile [62]. LACHESIS is a homolog of yeast PRP4, a kinase that influences mRNA splicing. AGO1 is the key effector endonuclease for multiple aspects of RNAi, including cleavage and translational inhibition of target messages via microRNA and trans-acting short interfering RNA (tasiRNA). Weak alleles of *AGO1* in *Arabidopsis* have drastic effects on leaf polarity [63]. KANADI is also a master regulator of leaf polarity, along with the auxin response factor ARF3 (a target of tasiRNA). The mRNA export factor homolog SDE5 also contributes positive PBS to the monocot clade, and is required for trans-acting siRNA accumulation [64], along with the RNA-dependent RNA polymerase RDR6, which was found to contribute positive PBS to the monocot clade in a smaller study of 17 taxa [65]. Mutants in the tasiRNA pathway disrupt the eponymous monocotyledonous embryo of rice, which displays radial asymmetry, far more severely than the symmetric dicotyledonous embryo of *Arabidopsis* [66], perhaps because tasiRNA act non-autonomously in *Arabidopsis*. Thus, it is hypothesized from this phylogenomic analysis that RNAi had a significant influence in the divergence of monocots and magnoliids plus eudicots from the ancestral angiosperm. This is an exciting hypothesis derived from the functional phylogenomic analysis of the seed plants, as we are increasingly aware of the importance of siRNAs and transcriptional gene silencing pathways of RNAi for plant evolution [67].

In perhaps the most important hypothesis derived from the phylogenomic approach we describe, the plant-specific RNA polymerase subunit NRPD2 contributes PBS to several nodes among lower plants, including conifers and *Marchantia*/moss, but especially to gymnosperms as a group, for which there are 21 steps of support, which is among the highest 2% of all genes with positive support for the gymnosperm clade. In *Arabidopsis,* NRPD2 is a subunit of both RNA polymerase IV and RNA polymerase V, both of which are required for 24nt siRNA biogenesis and for RNA-directed DNA methylation. Remarkably, 24nt small RNAs, which correspond to transposons and heterochromatic repeats in angiosperms, are absent from *Pinus contorta*, the only gymnosperm in which they have been examined [68]. They are nonetheless found in non-seed plants such as *Physcomitrella* and *Selaginella* [69]. Our phylogenomic analysis implicates NRPD2 in the loss of 24nt siRNA from gymnosperms that have very large unmethylated genomes [70], consistent with a loss of transposon control via siRNA.

In support of this derived hypothesis, high levels of maternal 24nt siRNA are found in the endosperm of developing *Arabidopsis* seeds in which NRPD2 is highly expressed [71]. In angiosperms, fertilization of both the egg and the central cell nucleus (double fertilization) lead to embryo and endosperm development, respectively, while in gymnosperms, the megagametophyte develops maternally without fertilization [71]. Interestingly, maternal mutants in *Arabidopsis* that disrupt this small RNA pathway are defective in transposon defense and develop unreduced gametophytes [72], the first step in maternal endosperm formation. We propose therefore that transposon-defense, mediated by small RNA, is responsible, in part, for the emergence of novel reproductive strategies within the flowering plants.

Along with NRPD2, a total of 297 genes in the phylogenomic tree provide PBS for the gymnosperm clade, while 407 genes provide PBS for the angiosperm clade, but the vast majority of the remaining ∼7,000 genes in this seed plant matrix provide support for individual nodes in the tree. These candidate genes lay the ground for new testable hypotheses concerning the evolutionary changes in function that may be of relevance in the astounding radiation of flowering plants, potentially underscoring Darwin's ‘abominable mystery’ of seed plant radiation [73].

In our effort to examine patterns of natural selection across the seed plants, we used established measures of synonymous (*dS*) and nonsynonymous (*dN*) nucleotide substitution rates – not in a gross fashion across the whole gene sequence, but rather on a codon-by-codon basis. The rate ratio *dN*/*dS* is a commonly used measure of selective pressure that has been expanded to incorporate sequence and codon evolution models, as well as branch rate variation [50]. *dS* is vulnerable to substitution saturation, and is not reliably estimated for very divergent taxa, even below the genus level [74]. In this study we are dealing with extant spermatophyte taxa that diverged near the Devonian–Carboniferous boundary at *ca.* 350 Ma, with their daughter groups diversifying after the Carboniferous (gymnosperms and conifers), and in the Jurassic in the last 200 million years (Myr) (angiosperms) [75], therefore substitution saturation is expected at synonymous substitutions. In an attempt to circumvent *dS* estimation issues, and thus undefined codon-specific *dN*/*dS* values, we allowed for the non-synonymous evolutionary rate to vary along the phylogeny, and more specifically, in the two subtrees united by the node where markedly high positive PBS scores where detected. We selected genus *Euphorbia* (angiosperms, eudicots, rosids, Malpighiales) as a case study. This is one of the most taxonomically rich plant genera likely encompassing ∼3000 species (http://www.plantsystematics.org) [76], that occupies habitats distributed worldwide and displays a marked degree of morphological and anatomical variation. The pantropical and very speciose family Euphorbiaceae (∼6300 species [76], 245 genera [77]) may have diverged from other Malpighiales in the Lower Cretaceous Aptian age around 119.4–101.1 Myr before present), but diversification within the family is much more recent, e.g. within Acalyphoideae within the last ∼70 Myr [78]. Note that Acalyphoideae is sister to the rest of the Euphorbiaceae including *Euphorbia* (see Figures 3 and 4 in [79]) that is itself nested inside Euphorbiaceae with an origin estimated at ∼38 Myr ago with most of its diversification having occurred in the interval 30–10 Myr before present (see Figure S1G in [80]). *Euphorbia* exhibited high positive PBS scores for 13 proteins involved in oxygen and radical detoxification (MIPS functional category 32.07.07). Codon-wise estimates of synonymous rates showed a highly positively skewed distribution within each gene, with discrepant median and mean values (*dS* across genes: median range  = 2.1–3.8, mean range  = 89–685, skewness  = 3.31–11.05), thus reinforcing our hesitation to use *dS* and subsequently *dN*/*dS* (Table S6). Gene-wide estimates of *dN*/*dS* [81] were well below 1.0 showing no evidence of the action of positive selection with rate ratio values ranging from 0.138 to 0.41 (Table S6). Contrasting *dN* between the subtree leading to *Euphorbia* and the “background” subtree, we found statistical evidence (*p*<0.05) of non-synonymous rate variation in 3 to 25 codons per gene. More specifically, we detected a general trend of non-synonymous rate acceleration in the subtree containing *Euphorbia*. A more complex picture became apparent in the case of 2 of those 13 genes (At1g76080 and At1g76080), where around half of the codons that showed significant evidence of *dN* rate change decelerated in the *Euphorbia*-containing clade. Detailed results are provided in Table S6.

## Discussion

Using a phylogenetic matrix with broad taxonomic sampling and gene representation, we are able to provide support for some of the more controversial topologies within plants, and in particular within the various hypotheses of gymnosperm evolution. From a phylogenomic perspective, we suggest hypotheses on genes and their evolutionary processes might be related to patterns in plant diversification. By focusing on the clade-specific variation of phylogenetic characters in a multi-gene matrix, we can determine the effect of individual genes or groups of genes within a particular gene category, on support metrics and their statistical correlation with functional processes of interest (such as seed development and gene silencing). Specifically, we can pinpoint the amino acid sequences that support individual branches in the tree. This enables us to investigate the genetic mechanisms that underlie the rapid radiation of the angiosperms and the persistence of the gymnosperms on a subset of candidate genes in follow-up studies. Our tree-based method, combined with maximum likelihood methods of non-synonymous and synonymous evolutionary rate variation, isolated 14 genes involved in oxygen and radical detoxification from one of the most speciose plant genera, exhibiting evidence for changes in selective pressure through non-synonymous rate heterogeneity. Most importantly, our functional phylogenomic method has shed light on the evolution of very large gymnosperm genomes, and on maternal endosperm development via the role of small interfering RNA in transposon defense and in asexual development.

In all, we demonstrate how a functional phylogenomic approach can be used to postulate hypotheses of gene function distributions and evolutionary mechanisms. Our framework sets the groundwork for future molecular biology, ecological genomics, and evolutionary development research, which will refine the hypothesized role of the genes we have identified herein. Furthermore, with the increasing amount of genomic sequence data available, we expect to see increased resolution of the seed plant tree, and more genes of importance to the evolution of major clades to be discovered using the phylogenomic methodology described herein.

## Materials and Methods

### Sequences and orthologs

Sequences were collected from the gene sets of 5 completely sequenced plant genomes and ESTs of 145 other plant species with at least 2,000 unigenes. The complete genomes include *Arabidopsis thaliana* (TAIR), *Oryza sativa* (JCVI), *Populus trichocarpa* (JGI), *Vitis vinifera* (Genoscope), and *Physcomitrella patens* (JGI). Unigenes were obtained from the TIGR Plant Transcript Assemblies (http://plantta.jcvi.org).

Orthology was determined using an extended OrthologID pipeline (Figure S1). The original OrthologID pipeline [21] only utilizes complete genomes in the generation of “guide trees” which are used to classify ESTs and determine their orthologs. However, the limited number of completely sequenced plant genomes to date would hamper the accuracy of EST placement when large numbers of ESTs from diverse plant species are classified into gene family trees with limited taxonomic representation. To alleviate this problem, we also included 17 extra species (ingroup and outgroup) with high number of ESTs in the generation of “guide trees”. These extra species represent a full spectrum of plant lineages and include: *Adiantum capillus-veneris*, *Aquilegia formosa*, *Amborella trichopoda*, *Ceratopteris richardii*, *Cichorium intybus*, *Coffea canephora*, *Gossypium hirsutum*, *Liriodendron tulipifera*, *Marchantia polymorpha*, *Medicago truncatula*, *Nuphar advena*, *Pinus taeda*, *Saruma henryi*, *Solanum tuberosum*, *Welwitschia mirabilis*, *Zamia fischeri*, and *Zingiber officinale*. As in the original OrthologID pipeline, gene families were clustered using a 1e-20 BLAST *E*-value cutoff, and were aligned with MAFFT [82] using three different sets of parameters with ambiguous regions culled. OrthologID generated guide trees from these gene families using parsimony. ESTs from all other species were then classified into gene families using the following stepwise shortest-tree method extrapolated from OrthologID: Each EST was identified with a gene family using BLAST. The complete set of ESTs that belong to a gene family were sorted in decreasing order of similarity according to their highest BLAST *e*-values against gene family members in the guide tree. Each EST was then inserted into the fixed guide tree in the aforementioned order. At every iteration the tree with the shortest length (most parsimonious) with respect to the guide tree is chosen. Finally, we determined sets of orthologs from the gene family tree by extracting the largest non-overlapping subsets of genes that are orthologous according to the topology. In cases where there is a 1-to-many or many-to-many ortholog relationship, each of the multiple orthologs is treated as equal and only a randomly selected one from each species is included in the matrix. In the >30%-matrix, multiple species of the same genus are represented by a single taxon. For each gene partition in this matrix, the gene sequence from the most ancestral of the species belonging to the same genus, as identified by the MP-full tree, is chosen to be the representative for that genus (taxon).

### Phylogenetic analysis

We assembled an alignment phylogenetic matrix of amino acid residues using the ortholog sets determined above and partitioned by gene. Only genes with at least four taxa present were included, resulting in a matrix with 22,833 partitions and 10,768,363 characters. Parsimony analysis was performed on the full alignment matrix and the >30%-representation genus alignment matrix using PAUP* v4b10 [83] and TNT [84]. The most efficient parsimony tree search strategy used the tree fusion method [85]: 100 jackknife resamplings (proportion = 0.3679) were searched with subtree pruning-regrafting holding two trees per resampling. The collected jackknife trees were then submitted to 100 rounds of tree fusion. Parsimony ratchet [86] also resulted in the same shortest trees (Figures S3 and S4). Node support was evaluated with 2,000 bootstrap pseudoreplicates and summarized on a 50% majority-rule consensus tree. Partitioned Bremer support (PBS) analysis was done on a submatrix that included only gene partitions with at least 10% taxon-representation. We use PBS to assess the direction of support (positive, negative, or neutral) of a particular gene to the various branches or nodes in a phylogeny. PBS is defined as follows: for a particular combined data set, a particular node (branch), and a particular data partition, PBS is the minimum number of character steps for that partition on the shortest topologies for the combined data set that do not contain that node, minus the minimum number of character steps for that partition on the shortest topologies for the combined data set that do contain that node [20]. Values for these metrics can be positive, zero or negative, and indicate the direction of support for the overall concatenated hypothesis: a positive PBS value indicates that the partition provides support for the node. Negative PBS means that the length of partition is shorter on an alternative tree (i.e. that partition provides contradictory evidence). The sum of PBS values for each data partition always equals Bremer Support for combined data [87]. We used TreeRot v3 [88] to generate PBS values for each partition-node pair.

We performed maximum likelihood (ML) inference of phylogeny on the >30% representation genus matrix using the fine-grained parallel Pthreads (POSIX Threads Library) [89] and MPI (Message Passing Interface) [90], [91] implementations of the 2009 development version of RAxML [92]. Our analysis represents the largest ML-based phylogenetic inference with respect to main memory requirements (89.2 GB) conducted to date. We employed both the JTT substitution matrix [93] with empirical amino acid residue frequencies (F), and the general time-reversible (GTR) substitution matrix [94] estimated directly from the genus-only alignment (Table S5). Both amino acid substitution models yielded the same overall topology. The JTT+F model was selected as the best-fit model based on its likelihood score among 22 models overall (11 with fixed residue frequencies and 11 with empirical residue frequencies calculated from the data in hand). We investigated the effect of the starting topology on the ML tree search and determined the best-scoring ML tree by employing ten random and ten randomized stepwise-addition MP trees in order to assess convergence to the final best ML topology, as well as the best single MP tree produced in our parsimony analysis. The way among-site rate heterogeneity was modeled had an impact on the final likelihood score; the CAT approximation model [95] with 25 per-site rate categories produced a better likelihood score than the Γ-distributed rate heterogeneity model with four discrete rates [96] in all cases where MP starting trees were used, while the GAMMA model performed better in 60% of the inferences when random starting trees were used (Table S4). Using the MP tree as a starting tree produced better likelihood scores for both rate heterogeneity models in conjunction with the GTR substitution model (logLik_CAT_  =  –35,802,562; logLik_GAMMA_  =  –35,802,416) (Table S4). Node support was quantified by means of 223 rapid non-parametric bootstrap pseudoreplicates (RBS) [97]. In order to determine if we conducted a sufficient number of RBS replicates we applied the novel bootstrap convergence test [98] implemented in RAxML *a posteriori* to our collection of 223 RBS trees. The Weighted and Frequency Criteria (WC and FC, respectively) [98] suggested that more than 74 and 122 replicates, respectively, would not induce significant changes on node support. Unlike majority-rule consensus trees, these support values indicate the percentage of pseudoreplicates in which the nodes of the best ML tree are present. Additional information on the ML analysis can be found in Text S1.

### Gene Ontology analysis

In order to functionally characterize the genes that are providing positive support to the phylogenetic tree, we identified the *Arabidopsis* orthologs of gene partitions with positive PBS at each node and determined GO [99] and MIPS [100] terms that are statistically overrepresented at each node. The analysis was performed using the BioMaps tool [101] available on VirtualPlant (http://www.virtualplant.org) with the hypergeometric statistics using the union of genes with positive PBS at all nodes as the background population, with a correction for multiple hypothesis testing. Terms with a *p*-value less than 0.05 were considered statistically significant. The Gene Ontology version of 31 May 2008 and the MIPS Functional Catalogue (FunCat) database version of 27 May 2008 were used. Given that we only include gene partitions with positive PBS in our functional GO term analysis, but do not use the actual PBS value in our statistical analysis, the use of a randomly selected ortholog in a gene partition in the case of multiple co-orthologs will likely have no effect on our results, except for the cases where the PBS values are very close to zero. In those scenarios, using a different co-ortholog may cause the PBS value to change from positive to non-positive, or vice versa, and therefore be excluded or included in the GO analysis. However, in a very large dataset, we expect the randomness to have a cancelling effect when we look at high-level GO categories that include many genes. Similarly, nodes with low support in our guide trees may affect orthology determination and subsequent GO term analysis. However, we expect the effect to be minimal due to our large sample size.

### Selection analysis

The extent of the pressure of natural selection was measured by estimating the ratio of the rate of nonsynonymous substitutions (*dN*) to the synonymous substitution rate (*dS*). A *dN*/*dS* rate value near one indicates neutral evolution, while deviations exceeding one are suggestive of positive selection, and positive values below one are considered to be evidence of purifying or negative selection as a result of strong structural or functional constraints at the protein level [102]. Functional protein-encoding genes tend to be subject to negative selection across their codons [103], therefore when only a few codons are positively selected [104] the measure of natural selection is averaged across all codons as a gene-wide *dN*/*dS* rate ratio. Our inference of episodic instances of positive selection becomes more powerful if, instead of averaging, we allow for *dN*/*dS* rate ratios to vary along the sequence alignment on a per-codon basis and across the phylogenetic tree [105], [106]. For this reason we estimated *dN*, *dS*, and *dN*/*dS* in a maximum likelihood framework as implemented in the latest development build of HyPhy v2.0 [107] (http://www.hyphy.org). Coding sequence evolution was modeled using the generalized Muse–Gaut (MG94) [108] model crossed with the Hasegawa–Kishino–Yano (HKY85) [109] nucleotide substitution model. The selective pressure at each codon site was quantified using the fixed effects likelihood (FEL) method [110] that estimates separately *dN* and *dS* rates for each codon and subsequently contrasts them through a likelihood ratio test (LRT). So as to avoid unpredictably biased *dN*/*dS* results because of saturation of the synonymous substitution rate *dS* across such a deep evolutionary timescale examined here, we chose to compare the variation of nonsynonymous rate *dN* along the phylogeny alongside gene-wide *dN*/*dS* estimates [81]. We selected a set of genes whose corresponding amino acid sequences exhibited strong, positive PBS (PBS>10) and belonged to the same MIPS term, such as 13 genes involved in oxygen and radical detoxification supporting consistently the node leading to the genus *Euphorbia* (*Arabidopsis* gene symbols: At1g65820, At1g76080, At2g47730, At1g64500, At3g54960, At3g15360, At4g33040, At1g20620, At4g31870, At1g19570, At3g27820, At5g23310, At2g31570). By allowing *dN* to vary in the two subsequent subtrees (*Euphorbia*-containing clade vs. the rest of the tree), we discounted possible *dS* biases and examined whether there is statistical evidence through a LRT for a change in nonsynonymous rates.

### Computing

We performed maximum parsimony (MP) and Bremer support analysis on a 64-node Linux cluster at NYU. MP bootstrap analysis was performed on the 2,000-node BlueHelix HPCC facility at CSHL. In order to analyze this challenging dataset under maximum likelihood (ML) we used several clusters, multi-core nodes, and supercomputers: the Woodcrest Cluster at the Regionales Rechenzentrum Erlangen in Germany (868 Intel Woodcrest cores, Infiniband interconnect), the Infiniband Cluster at the Technical University of Munich (128 AMD Opteron cores, Infiniband interconnect), the AMD Barcelona multi-core nodes at the Swiss Federal Institute of Technology in Lausanne (2 16-core AMD Barcelona nodes), and the SGI ALTIX 4700 supercomputer at the Leibniz Rechenzentrum in Munich (8192 Intel Itanium cores, custom interconnect). The selection ML analysis was carried out on an Apple Mac Pro 12-core with Intel Xeon 2.66 GHz processors and 8 GB of RAM (1333 MHz DDR3) running 20 processes for each inference.

The complete phylogenomic matrix and trees are available at the *BIGPLANT* website (http://nypg.bio.nyu.edu).

## Supporting Information

Figure S1 Extended OrthologID pipeline. (A) Complete genomes and a selected sets of species with large number of ESTs are clustered into gene families. These families are aligned and phylogenetic trees created. Orthologs are determined according to the phylogenies of these gene families. (B) Sets of orthologs are determined from a gene family tree in the following manner: as we move up from the taxa towards the root of the tree, sets of genes are gathered at each node, until the sets of species of two descendent nodes overlap. This indicated we encounter a duplication event, in which case we output the two sets of orthologs separately as they are not orthologous to each other. In the figure, A, B, and C are species. Two sets of orthologs are identified: {A1, B1, C1} and {A2, A3, B2, C2}.
(EPS)

Figure S2 Cumulative distribution of gene partitions by taxon density in the >30% matrix. The rate of increase in gene partitions decreases exponentially with the number of taxa represented.
(EPS)

Figure S3 Maximum parsimony simultaneous analysis tree of 150 taxa and 22,833 genes (MP-full). Support values are based on 2,000 bootstrap pseudoreplicates.
(EPS)

Figure S4 Maximum parsimony simultaneous analysis tree of the genus-only matrix with 101 taxa and at least 30% representation per partition (MP-30). Support values are based on 2,000 bootstrap pseudo-replicates. Nodes with overrepresented GO/MIPS terms have numeric labels that match the node numbers in Table S3.
(EPS)

Figure S5 Our ML-30 tree is in broad agreement with the APG III tree. Relationships among major clades are identical, with the main exception of the magnoliids as shown here.
(EPS)

Figure S6 Impact on character optimization due to different topologies. Depending on the topology, a single character can be interpreted as an apomorphy in one group and a plesiomorphy in another, or a result of convergence and independently derived apomorphies. (A) Simple and compound strobilus. Photos of cycad megasporophylls (right), *Liriodendrum tulipifera* (tulip) flower (upper left) and *Welwitschia mirabilis* seed cones (lower left) illustrate seed-bearing sporophylls, a gynoecium and ovulate strobili respectively. (B) Absence and presence of motile sperm. Photo of motile spermatozoid of *Zamia pumila* is shown.
(EPS)

Figure S7 Proportion of the number of genes with a positive PBS (PPBS) from the total of genes per GO and MIPs terms. In (A) all nodes in our tree, and (B) nodes highlighted in Figure 2 (GO and MIPs terms are combined), shown here is *Euphorbia* and sister taxa 19; (*Cucumis*/*Juglans regia*) 31; (*Capsicum annum/Solanum*) 52; (*Nicotiana* (*Capsicum annum/Solanum*) 53; the split of the Caryophillids 65; the split of *Acorus* 94; and the Monocots clade 95.
(EPS)

Table S1 List of 150 plant species, sequence sources, and the number of genes represented in the full matrix.
(DOC)

Table S2 Number of genes with identifiable *Arabidopsis* orthologs in the full matrix for each GO Slim category. Each gene may belong to more than one category.
(DOC)

Table S3 Complete list of overrepresented GO/MIPS terms on the MP-30 tree. Nodes are numbered as in Figure S4.
(DOC)

Table S4 Log-likelihood scores for inferences based on the CAT and GAMMA rate heterogeneity models from different starting trees under the GTR substitution matrix. Inferences that yielded better likelihood scores in CAT-GAMMA comparisons are shaded for clarity.
(DOC)

Table S5 GreenREV – a dedicated amino acid general time-reversible (GTR) substitution matrix estimated from our dataset. Rectangular symmetric GTR substitution rate matrix, where amino acids are ordered alphabetically according to their three-letter code. The bottom line contains the empirical amino acid frequencies in the same order. This can be used as a user-defined amino acid substitution matrix in RAxML using the -P option. See file GreenREV.txt.
(TXT)

Table S6 Selection analysis results for the *Euphorbia* candidate genes. Values given for 13 genes whose amino acid sequence showed strong, positive PBS (PBS>10) and belonged to the same MIPS term.
(DOC)

Text S1 Supporting Methods.
(PDF)
