# Supplementary material for: A Functional Phylogenomic View of the Seed Plants
Source: PLoS Genet. 2011 Dec 15;7(12):e1002411. doi: 10.1371/journal.pgen.1002411 (PMC3240601; doi:10.1371/journal.pgen.1002411)
Supplement: Table S1 — List of 150 plant species, sequence sources, and the number of genes represented in the full matrix. (DOC) [file pgen.1002411.s008.doc]

**Table S1. List of 150 plant species, sequence sources, and the number of genes represented in the full matrix.**

| **Species** | **Sources** | **Number of genes in alignment matrix** |
| --- | --- | --- |
| *Acorus americanus* | PlantTA | 2,084 |
| *Adiantum capillus-veneris* | PlantTA | 473 |
| *Aegilops speltoides* | PlantTA | 709 |
| *Agrostis capillaris* | PlantTA | 1,057 |
| *Agrostis stolonifera* | PlantTA | 625 |
| *Allium cepa* | PlantTA | 3,879 |
| *Amborella trichopoda* | PlantTA | 1,090 |
| *Ananas comosus* | PlantTA | 1,167 |
| *Antirrhinum majus* | PlantTA | 4,660 |
| *Aquilegia formosa* | PlantTA | 4,227 |
| *Arabidopsis thaliana* | TAIR | 7,537 |
| *Arachis hypogaea* | PlantTA | 1,169 |
| *Asparagus officinalis* | PlantTA | 1,968 |
| *Avena sativa* | PlantTA | 1,132 |
| *Beta vulgaris* | PlantTA | 4,632 |
| *Brachypodium distachyon* | PlantTA | 2,819 |
| *Brassica napus* | PlantTA | 5,866 |
| *Brassica oleracea* | PlantTA | 3,514 |
| *Brassica rapa* | PlantTA | 3,298 |
| *Bruguiera gymnorrhiza* | PlantTA | 1,948 |
| *Capsicum annuum* | PlantTA | 4,274 |
| *Ceratopteris richardii* | PlantTA | 430 |
| *Chamaecyparis obtusa* | PlantTA | 1,127 |
| *Cichorium intybus* | PlantTA | 5,884 |
| *Citrus aurantium* | PlantTA | 1,110 |
| *Citrus clementina* | PlantTA | 6,059 |
| *Citrus reshni* | PlantTA | 694 |
| *Citrus reticulata* | PlantTA | 857 |
| *Citrus sinensis* | PlantTA | 6,769 |
| *Coffea canephora* | PlantTA | 3,431 |
| *Cryptomeria japonica* | PlantTA | 2,099 |
| *Cucumis melo* | PlantTA | 1,229 |
| *Cucumis sativus* | PlantTA | 1,257 |
| *Curcuma longa* | PlantTA | 1,548 |
| *Cycas rumphii* | CSHL/PlantTA | 1,096 |
| *Cyclamen persicum* | PlantTA | 358 |
| *Eragrostis tef* | PlantTA | 433 |
| *Eschscholzia californica* | PlantTA | 2,071 |
| *Euphorbia esula* | PlantTA | 7,602 |
| *Euphorbia tirucalli* | PlantTA | 2,249 |
| *Festuca arundinacea* | PlantTA | 4,114 |
| *Fragaria vesca* | PlantTA | 3,198 |
| *Gerbera hybrid* | PlantTA | 2,540 |
| *Ginkgo biloba* | CSHL/PlantTA | 875 |
| *Glycine max* | PlantTA | 10,071 |
| *Glycine soja* | PlantTA | 3,298 |
| *Gnetum gnemon* | CSHL/PlantTA | 528 |
| *Gossypium arboreum* | PlantTA | 3,901 |
| *Gossypium hirsutum* | PlantTA | 5,641 |
| *Gossypium raimondii* | PlantTA | 5,622 |
| *Hedyotis centranthoides* | PlantTA | 1,084 |
| *Hedyotis terminalis* | PlantTA | 990 |
| *Helianthus annuus* | PlantTA | 6,162 |
| *Helianthus argophyllus* | PlantTA | 5,327 |
| *Helianthus exilis* | PlantTA | 6,110 |
| *Helianthus paradoxus* | PlantTA | 1,194 |
| *Helianthus petiolaris* | PlantTA | 4,127 |
| *Hevea brasiliensis* | PlantTA | 1,390 |
| *Hordeum vulgare* | PlantTA | 7,842 |
| *Ipomoea batatas* | PlantTA | 2,173 |
| *Ipomoea nil* | PlantTA | 5,955 |
| *Juglans regia* | PlantTA | 980 |
| *Lactuca perennis* | PlantTA | 4,219 |
| *Lactuca saligna* | PlantTA | 4,580 |
| *Lactuca sativa* | PlantTA | 7,657 |
| *Lactuca serriola* | PlantTA | 6,185 |
| *Lactuca virosa* | PlantTA | 4,258 |
| *Linum usitatissimum* | PlantTA | 1,046 |
| *Liriodendron tulipifera* | PlantTA | 1,208 |
| *Lolium multiflorum* | PlantTA | 936 |
| *Lolium temulentum* | PlantTA | 937 |
| *Lotus japonicus* | PlantTA | 6,488 |
| *Lycoris longituba* | PlantTA | 444 |
| *Manihot esculenta* | PlantTA | 2,472 |
| *Marchantia polymorpha* | PlantTA | 961 |
| *Medicago sativa* | PlantTA | 1,100 |
| *Medicago truncatula* | PlantTA | 6,203 |
| *Mesembryanthemum crystallinum* | PlantTA | 3,029 |
| *Mimulus guttatus* | PlantTA | 1,497 |
| *Nicotiana benthamiana* | PlantTA | 2,371 |
| *Nicotiana sylvestris* | PlantTA | 2,019 |
| *Nicotiana tabacum* | PlantTA | 6,330 |
| *Nuphar advena* | PlantTA | 1,112 |
| *Ocimum basilicum* | PlantTA | 2,097 |
| *Oryza minuta* | PlantTA | 1,203 |
| *Oryza sativa* | JCVI | 7,295 |
| *Panax ginseng* | PlantTA | 1,406 |
| *Panicum virgatum* | PlantTA | 1,619 |
| *Pennisetum glaucum* | PlantTA | 274 |
| *Persea americana* | PlantTA | 1,936 |
| *Phalaenopsis equestris* | PlantTA | 927 |
| *Phaseolus coccineus* | PlantTA | 2,332 |
| *Phaseolus vulgaris* | PlantTA | 3,886 |
| *Physcomitrella patens* | JGI | 1,993 |
| *Picea abies* | PlantTA | 1,189 |
| *Picea glauca* | PlantTA | 5,434 |
| *Picea sitchensis* | PlantTA | 4,135 |
| *Pinus pinaster* | PlantTA | 3,148 |
| *Pinus taeda* | PlantTA | 4,850 |
| *Pisum sativum* | PlantTA | 838 |
| *Poncirus trifoliata* | PlantTA | 3,546 |
| *Populus deltoides* | PlantTA | 2,918 |
| *Populus euphratica* | PlantTA | 2,993 |
| *Populus nigra* | PlantTA | 1,248 |
| *Populus tremula* | PlantTA | 3,524 |
| *Populus tremuloides* | PlantTA | 1,775 |
| *Populus trichocarpa* | JGI | 8,218 |
| *Prunus armeniaca* | PlantTA | 1,946 |
| *Prunus persica* | PlantTA | 5,562 |
| *Pseudotsuga menziesii* | PlantTA | 942 |
| *Puccinellia tenuiflora* | PlantTA | 173 |
| *Ricinus communis* | PlantTA | 3,892 |
| *Robinia pseudoacacia* | PlantTA | 405 |
| *Rosa hybrid* | PlantTA | 1,319 |
| *Saccharum hybrid* | PlantTA | 1,536 |
| *Saccharum officinarum* | PlantTA | 8,176 |
| *Salvia miltiorrhiza* | PlantTA | 1,500 |
| *Saruma henryi* | PlantTA | 1,588 |
| *Secale cereale* | PlantTA | 1,173 |
| *Solanum chacoense* | PlantTA | 2,406 |
| *Solanum habrochaites* | PlantTA | 1,510 |
| *Solanum lycopersicum* | PlantTA | 8,616 |
| *Solanum pennellii* | PlantTA | 1,374 |
| *Solanum tuberosum* | PlantTA | 6,597 |
| *Sorghum bicolor* | PlantTA | 6,597 |
| *Sorghum propinquum* | PlantTA | 2,088 |
| *Stevia rebaudiana* | PlantTA | 1,489 |
| *Tamarix androssowii* | PlantTA | 201 |
| *Taraxacum kok-saghyz* | PlantTA | 1,551 |
| *Taraxacum officinale* | PlantTA | 4,818 |
| *Thellungiella salsuginea* | PlantTA | 1,775 |
| *Theobroma cacao* | PlantTA | 843 |
| *Thlaspi caerulescens* | PlantTA | 928 |
| *Tortula ruralis* | PlantTA | 487 |
| *Trifolium pratense* | PlantTA | 3,883 |
| *Triphysaria versicolor* | PlantTA | 1,356 |
| *Triticum aestivum* | PlantTA | 9,214 |
| *Triticum monococcum* | PlantTA | 1,560 |
| *Triticum turgidum* | PlantTA | 1,440 |
| *Vaccinium corymbosum* | PlantTA | 1,193 |
| *Vitis hybrid* | PlantTA | 624 |
| *Vitis shuttleworthii* | PlantTA | 2,230 |
| *Vitis vinifera* | Genoscope | 6,633 |
| *Welwitschia mirabilis* | PlantTA | 1,337 |
| *Zamia fischeri* | CSHL/PlantTA | 746 |
| *Zantedeschia aethiopica* | PlantTA | 554 |
| *Zea mays* | PlantTA | 8,783 |
| *Zea mays B73* | PlantTA | 7,839 |
| *Zingiber officinale* | PlantTA | 2,182 |
| *Zinnia elegans* | PlantTA | 3,451 |

CSHL – Cold Spring Harbor Laboratory, USA

Genoscope – Centre National de Séquençage, France

JGI – U.S. Department of Energy Joint Genome Institute

JCVI – J. Craig Venter Institute, USA

TAIR – The Arabidopsis Information Resource ([www.arabidopsis.org](http://www.arabidopsis.org/))

PlantTA – TIGR Plant Transcript Assemblies ([plantta.jcvi.org](http://plantta.jcvi.org/))
