# Supplementary material for: A Functional Phylogenomic View of the Seed Plants
Source: PLoS Genet. 2011 Dec 15;7(12):e1002411. doi: 10.1371/journal.pgen.1002411 (PMC3240601; doi:10.1371/journal.pgen.1002411)
Supplement: Table S2 — Number of genes with identifiable Arabidopsis orthologs in the full matrix for each GO Slim category. Each gene may belong to more than one category. (DOC) [file pgen.1002411.s009.doc]

**Table S2. Number of genes with identifiable *Arabidopsis* orthologs in the full matrix for each GO Slim category.** Each gene may belong to more than one category.

| **GO Slim term** | **Number of genes** |
| --- | --- |
| *Molecular Function* | |
| hydrolase activity | 644 |
| kinase activity | 219 |
| transferase activity | 617 |
| other enzyme activity | 936 |
| transcription factor activity | 409 |
| DNA or RNA binding | 573 |
| nucleic acid binding | 205 |
| nucleotide binding | 393 |
| protein binding | 667 |
| receptor binding or activity | 29 |
| other binding | 786 |
| structural molecule activity | 163 |
| transporter activity | 361 |
| other molecular functions | 325 |
| unknown molecular functions | 1,986 |
| *Biological Process* | |
| developmental processes | 496 |
| transport | 530 |
| signal transduction | 189 |
| cell organization and biogenesis | 382 |
| other cellular processes | 2,632 |
| DNA or RNA metabolism | 171 |
| protein metabolism | 799 |
| electron transport or energy pathways | 92 |
| transcription | 449 |
| other metabolic processes | 2,476 |
| response to abiotic or biotic stimulus | 483 |
| response to stress | 453 |
| other biological processes | 420 |
| unknown biological process | 2,499 |
| *Cellular Component* | |
| mitochondria | 405 |
| chloroplast | 1,338 |
| plastid | 544 |
| ribosome | 148 |
| cytosol | 160 |
| endoplasmic reticulum | 137 |
| Golgi apparatus | 90 |
| other cytoplasmic components | 1,182 |
| nucleus | 821 |
| other intracellular components | 1,536 |
| plasma membrane | 611 |
| other membranes | 1,013 |
| extracellular | 78 |
| cell wall | 102 |
| other cellular components | 570 |
| unknown cellular components | 1,775 |

GO Slim annotation source: TAIR ([http://arabidopsis.org](http://arabidopsis.org/))
