# Supplementary material for: A Functional Phylogenomic View of the Seed Plants
Source: PLoS Genet. 2011 Dec 15;7(12):e1002411. doi: 10.1371/journal.pgen.1002411 (PMC3240601; doi:10.1371/journal.pgen.1002411)
Supplement: Table S3 — Complete list of overrepresented GO/MIPS terms on the MP-30 tree. Nodes are numbered as in Figure S4. (DOC) [file pgen.1002411.s010.doc]

**Table S3. Complete list of overrepresented GO/MIPS terms on the MP-30 tree.** Nodes are numbered as in Figure S4.

| **Go/MIPS Term** | **Observed Frequency (%)** | **Expected Frequency (%)** | ***p*-value** | **Gene ID of *Arabidopsis* orthologs in alignment matrix** |
| --- | --- | --- | --- | --- |
| Node 1 |  |  |  |  |
| sulfur compound biosynthetic process | 4.1 | 0.1 | 0.081 | At4g23100 At4g33030 |
| Node 2 |  |  |  |  |
| cellular biosynthetic process | 22.6 | 6.8 | 0.003 | At4g33760 At4g11120 At3g46100 At4g33030 At2g40570 At1g57770 At5g10870 At3g15870 At4g23100 At3g46940 At3g25920 At3g61530 At4g13050 At3g24030 |
| Node 6 |  |  |  |  |
| cellular biosynthetic process | 16.1 | 6.8 | 0.093 | At4g33250 At5g23535 At5g66120 At5g11750 At3g14110 At5g58240 At5g24510 At3g63490 At3g46940 At5g14320 At3g63410 At2g35500 At1g32100 At5g13510 At3g13120 |
| cytoplasm | 14 | 4 | 0.006 | At4g33250 At5g24510 At5g13510 At1g18800 At5g14320 At5g10480 At5g11750 At3g01640 At3g13120 At2g38130 At3g63490 At2g40120 At2g26350 |
| ribosome biogenesis | 7.5 | 1.5 | 0.03 | At5g14320 At5g11750 At3g13120 At3g63490 At5g24510 At5g23535 At5g13510 |
| translation | 8.6 | 2 | 0.043 | At4g33250 At5g24510 At5g23535 At5g13510 At5g14320 At3g13120 At5g11750 At3g63490 |
| Node 7 |  |  |  |  |
| cellular macromolecule metabolic process | 23.7 | 10.3 | 0.005 | At3g05230 At1g02560 At5g23535 At3g60210 At4g17040 At2g43030 At5g24510 At5g10480 At1g69830 At5g13510 At5g57860 At1g12410 At1g06900 At2g40120 At1g79850 At1g05820 At3g46920 At5g60160 At5g64050 At1g55670 At2g07340 At3g54210 At3g13120 |
| cellular protein metabolic process | 22.7 | 9.7 | 0.06 | At3g05230 At1g02560 At5g23535 At3g60210 At4g17040 At2g43030 At5g24510 At5g10480 At5g13510 At5g57860 At1g12410 At1g06900 At2g40120 At1g79850 At1g05820 At3g46920 At5g60160 At5g64050 At1g55670 At2g07340 At3g54210 At3g13120 |
| protein metabolic process | 22.7 | 10.1 | 0.011 | At3g05230 At1g02560 At5g23535 At3g60210 At4g17040 At2g43030 At5g24510 At5g10480 At5g13510 At5g57860 At1g12410 At1g06900 At2g40120 At1g79850 At1g05820 At3g46920 At5g60160 At5g64050 At1g55670 At2g07340 At3g54210 At3g13120 |
| ATP-dependent proteolysis | 3.1 | 0.2 | 0.058 | At1g02560 At4g17040 At1g12410 |
| ribosome biogenesis | 8.2 | 1.5 | 0.005 | At1g79850 At2g43030 At5g24510 At5g23535 At5g13510 At3g13120 At5g08180 At3g54210 |
| protein processing (proteolytic) | 4.1 | 0.3 | 0.014 | At3g05230 At1g12410 At1g02560 At4g17040 |
| translation | 8.2 | 2 | 0.05 | At1g79850 At2g43030 At5g24510 At5g23535 At5g13510 At3g13120 At5g08180 At3g54210 |
| protein synthesis | 9.3 | 2.8 | 0.087 | At1g79850 At2g43030 At5g64050 At5g24510 At5g23535 At5g13510 At3g13120 At5g08180 At3g54210 |
| Node 8 |  |  |  |  |
| cellular macromolecule metabolic process | 23 | 10.3 | 0.009 | At3g05230 At1g02560 At5g23535 At3g60210 At4g17040 At2g43030 At5g24510 At5g10480 At1g69830 At5g13510 At5g57860 At1g12410 At1g06900 At2g40120 At1g79850 At1g05820 At3g46920 At5g60160 At5g64050 At1g55670 At2g07340 At3g54210 At3g13120 |
| cellular protein metabolic process | 22 | 9.7 | 0.011 | At3g05230 At1g02560 At5g23535 At3g60210 At4g17040 At2g43030 At5g24510 At5g10480 At5g13510 At5g57860 At1g12410 At1g06900 At2g40120 At1g79850 At1g05820 At3g46920 At5g60160 At5g64050 At1g55670 At2g07340 At3g54210 At3g13120 |
| protein metabolic process | 22 | 10.1 | 0.019 | At3g05230 At1g02560 At5g23535 At3g60210 At4g17040 At2g43030 At5g24510 At5g10480 At5g13510 At5g57860 At1g12410 At1g06900 At2g40120 At1g79850 At1g05820 At3g46920 At5g60160 At5g64050 At1g55670 At2g07340 At3g54210 At3g13120 |
| ATP-dependent proteolysis | 3 | 0.2 | 0.067 | At1g02560 At4g17040 At1g12410 |
| ribosome biogenesis | 8 | 1.5 | 0.007 | At1g79850 At2g43030 At5g24510 At5g23535 At5g13510 At3g13120 At5g08180 At3g54210 |
| protein processing (proteolytic) | 4 | 0.3 | 0.017 | At3g05230 At1g12410 At1g02560 At4g17040 |
| protein synthesis | 10 | 2.8 | 0.028 | At1g79850 At2g43030 At5g64050 At5g24510 At5g23535 At5g13510 At3g13120 At5g16710 At5g08180 At3g54210 |
| translation | 8 | 2 | 0.066 | At1g79850 At2g43030 At5g24510 At5g23535 At5g13510 At3g13120 At5g08180 At3g54210 |
| Node 14 |  |  |  |  |
| electron transport | 20 | 2.7 | 0.043 | At1g10960 At1g20340 At3g15360 At5g51010 |
| Node 15 |  |  |  |  |
| sulfur compound catabolic process | 4.9 | 0 | 0.002 | At4g29210 At1g64660 |
| photosynthesis | 4.9 | 0.2 | 0.085 | At4g04020 At4g14690 |
| Node 18 |  |  |  |  |
| response to gibberellin stimulus | 1.9 | 0.3 | 0.019 | At5g06650 At5g11260 At3g16350 At3g47620 At4g25000 At2g37630 |
| Node 19 |  |  |  |  |
| oxygen and radical detoxification | 3.9 | 0.9 | 0.001 | At1g76080 At2g31570 At5g39950 At4g33040 At1g20620 At1g19570 At2g47730 At3g54960 At1g65820 At5g23310 At1g64500 At4g31870 At3g15360 At3g27820 |
| detoxification | 3.9 | 1 | 0.001 | At1g76080 At2g31570 At5g39950 At4g33040 At1g20620 At1g19570 At2g47730 At3g54960 At1g65820 At5g23310 At1g64500 At4g31870 At3g15360 At3g27820 |
| cell rescue, defense and virulence | 9.5 | 4.3 | 0.001 | At2g31570 At5g39950 At4g33040 At1g19570 At3g23830 At4g21320 At1g35680 At1g64500 At3g15360 At4g31870 At1g11360 At5g37670 At5g25610 At2g47730 At5g23310 At1g65820 At3g27820 At3g59380 At3g62550 At1g01520 At1g20620 At4g02580 At2g16500 At2g37630 At3g22200 At3g16350 At1g76080 At1g06460 At4g02600 At2g21620 At2g25930 At3g54960 At4g10250 At4g00860 |
| translation | 5.3 | 2 | 0.016 | At5g18110 At1g71350 At5g41520 At1g35680 At1g70600 At2g42710 At5g46160 At2g19720 At5g53070 At2g45730 At1g48350 At5g38290 At4g30690 At1g79850 At1g18540 At5g54600 At3g27850 At3g08740 At1g75350 |
| cytoplasm | 7.8 | 4 | 0.074 | At5g18110 At2g31570 At5g47120 At5g41520 At5g39950 At4g14800 At1g35680 At1g70600 At3g07720 At4g08170 At2g42710 At5g67530 At5g57950 At5g46160 At1g18800 At4g18930 At2g43640 At2g19720 At5g53070 At1g11860 At1g48350 At1g79850 At1g18540 At4g36060 At1g56450 At3g42850 At2g16950 At1g75350 |
| Node 21 |  |  |  |  |
| cellular transport, transport facilitation and transport routes | 16.9 | 9.5 | 0.059 | At2g01170 At3g15990 At2g17500 At1g60070 At5g09930 At4g26590 At2g28520 At2g47830 At1g05200 At4g30720 At2g43950 At3g51840 At5g56160 At3g14120 At1g01910 At1g19780 At1g04980 At4g24170 At3g14150 At1g03000 At4g28710 At1g14670 At1g08960 At3g58730 At5g06580 At1g32350 At4g25650 At1g32500 At5g41000 At1g73860 At4g19380 At3g16110 At5g12860 At3g16270 At2g31530 |
| Node 22 |  |  |  |  |
| toxin metabolic process | 4.2 | 0.1 | 0.029 | At5g62480 At1g10360 |
| toxin catabolic process | 4.2 | 0.1 | 0.029 | At5g62480 At1g10360 |
| response to desiccation | 4.2 | 0.1 | 0.043 | At5g25610 At2g44060 |
| metabolism of toxins/drugs | 4.2 | 0.1 | 0.052 | At1g10360 At5g62480 |
| energy conversion and regeneration | 4.2 | 0.1 | 0.052 | At5g13450 At4g09650 |
| energy generation (e.g. ATP synthase) | 4.2 | 0.1 | 0.052 | At5g13450 At4g09650 |
| Node 29 |  |  |  |  |
| cellular process | 56.6 | 36.8 | 0.001 | At4g14690 At4g24770 At5g42850 At5g39510 At1g20050 At4g22720 At4g18040 At1g09340 At1g12410 At5g55170 At2g43430 At5g42220 At3g02870 At3g60820 At2g18040 At5g13710 At2g27730 At1g09130 At1g06200 At3g27820 At4g03280 At5g13930 At2g28900 At4g39980 At2g31490 At2g37660 At2g31440 At5g40930 At2g47730 At5g27450 At3g17760 At1g18540 At5g06360 At5g61170 At4g21550 At1g75390 At4g04020 At1g69830 At4g31540 At3g03860 At2g24200 At5g48560 At1g68940 At2g35795 At1g09795 At1g32220 At3g55005 At3g06310 At3g66654 At4g20410 At3g51770 At2g41500 At5g41520 At5g66860 At3g21865 At1g10360 At2g45200 At1g60970 At1g79850 At1g35620 At2g44350 At1g18800 At3g55360 At5g54310 |
| metabolic process | 49.6 | 34.4 | 0.055 | At4g21550 At4g14690 At4g24770 At1g75390 At4g04020 At1g20050 At4g22720 At4g18040 At1g69830 At1g12410 At5g48560 At2g24200 At1g65930 At5g55170 At2g43430 At5g42220 At1g68940 At3g02870 At2g35795 At3g60820 At5g13710 At5g59480 At1g76150 At1g09795 At2g27730 At1g09130 At1g32220 At1g06200 At3g06310 At3g66654 At3g27820 At4g03280 At5g13930 At1g15410 At4g39980 At2g31490 At2g37660 At2g31440 At3g51770 At2g41500 At5g41520 At2g47730 At5g66860 At5g27450 At3g17760 At1g10360 At1g18540 At5g06360 At3g01850 At5g61170 At1g11840 At1g79850 At2g32090 At2g44350 At3g55360 At1g18800 |
| endoplasmic reticulum | 5.3 | 0.7 | 0.014 | At4g20410 At5g13930 At3g60820 At1g20050 At3g55005 At3g55360 |
| cytoplasm | 11.5 | 4 | 0.052 | At1g10360 At5g41520 At1g79850 At3g60820 At1g18540 At2g41430 At5g06360 At5g61170 At1g18800 At4g18040 At5g27450 At1g68940 At2g43430 |
| Node 31 |  |  |  |  |
| response to oxidative stress | 9.5 | 0.6 | 0.003 | At4g31870 At5g37510 At2g16500 At4g02580 |
| oxidative stress response | 9.5 | 0.5 | 0.002 | At4g02580 At4g31870 At5g37510 At2g16500 |
| oxygen and radical detoxification | 9.5 | 0.9 | 0.027 | At3g54660 At4g31870 At5g16710 At4g37200 |
| detoxification | 9.5 | 1 | 0.034 | At3g54660 At4g31870 At5g16710 At4g37200 |
| cell rescue, defense and virulence | 16.7 | 4.3 | 0.084 | At4g02580 At3g54660 At4g31870 At5g37510 At2g16500 At5g16710 At4g37200 |
| Node 33 |  |  |  |  |
| protein import into chloroplast stroma | 5.5 | 0.1 | 0 | At2g28900 At2g15290 At5g16620 |
| protein targeting to chloroplast | 5.5 | 0.1 | 0.002 | At2g28900 At2g15290 At5g16620 |
| protein import | 5.5 | 0.3 | 0.037 | At2g28900 At2g15290 At5g16620 |
| ribosomal proteins | 7.3 | 0.7 | 0.024 | At5g54600 At5g41520 At5g14320 At4g10450 |
| Node 34 |  |  |  |  |
| metabolic compound salvage | 4.9 | 0.2 | 0.091 | At4g16450 At2g22570 |
| Node 36 |  |  |  |  |
| pollen-pistil interaction | 3.2 | 0 | 0.008 | At3g22200 At1g11300 |
| Node 37 |  |  |  |  |
| pollen-pistil interaction | 2.3 | 0 | 0.018 | At3g22200 At1g11300 |
| post Golgi transport | 2.3 | 0.1 | 0.061 | At1g28490 At5g39510 |
| Node 39 |  |  |  |  |
| ribosome biogenesis | 4.4 | 1.5 | 0.02 | At4g28360 At5g67510 At1g79850 At1g72370 At5g41520 At2g43030 At1g05190 At3g57000 At5g23535 At3g53890 At1g32990 At5g14320 At3g63490 At5g08180 At1g75350 |
| protein synthesis | 6.4 | 2.8 | 0.026 | At4g28360 At1g72370 At5g41520 At2g43030 At5g23535 At3g01800 At3g57190 At3g53890 At3g08010 At5g14320 At5g49030 At3g49100 At5g67510 At1g79850 At1g05190 At5g64050 At3g57000 At1g32990 At3g63490 At1g49760 At5g08180 At1g75350 |
| translation | 5.3 | 2 | 0.03 | At4g28360 At1g72370 At5g41520 At2g43030 At5g23535 At3g01800 At3g57190 At3g53890 At3g08010 At5g14320 At3g49100 At5g67510 At1g79850 At1g05190 At3g63490 At1g49760 At5g08180 At1g75350 |
| Node 44 |  |  |  |  |
| biosynthetic process | 100 | 14.4 | 0.042 | At4g33250 At1g32100 At3g18380 |
| nucleic acid binding | 66.7 | 9.4 | 0.074 | At4g33250 At3g18380 |
| Node 45 |  |  |  |  |
| response to far red light | 5.3 | 0.1 | 0.02 | At4g14690 At5g24120 |
| response to red light | 5.3 | 0.1 | 0.055 | At4g14690 At5g24120 |
| photosynthesis | 5.3 | 0.2 | 0.082 | At4g04020 At4g14690 |
| Node 46 |  |  |  |  |
| ATP-dependent proteolysis | 1.8 | 0.2 | 0.029 | At2g25740 At1g09130 At1g02560 At4g17040 At1g12410 |
| response to jasmonic acid stimulus | 2.2 | 0.4 | 0.091 | At2g28900 At4g23100 At3g23240 At5g64930 At4g37760 At2g37630 |
| cell rescue, defense and virulence | 9.4 | 4.3 | 0.016 | At3g51780 At1g01520 At1g20340 At5g39950 At5g50200 At4g23100 At1g44575 At1g35620 At3g15360 At2g18260 At5g22360 At3g05210 At2g37630 At5g16710 At2g16500 At3g23240 At1g06460 At5g25610 At3g52960 At5g54960 At5g49480 At5g64930 At3g27820 At2g28900 At4g37760 At3g01520 |
| photosynthesis | 1.4 | 0.2 | 0.084 | At2g26500 At1g14150 At1g55670 At2g21970 |
| Node 50 |  |  |  |  |
| amino acid biosynthetic process | 8.5 | 0.8 | 0.034 | At5g16290 At5g66120 At5g10870 At1g18640 |
| amino acid and derivative metabolic process | 12.8 | 2.3 | 0.043 | At5g16290 At1g11860 At5g66120 At5g10870 At1g18640 At1g32100 |
| amine biosynthetic process | 8.5 | 0.9 | 0.046 | At5g16290 At5g66120 At5g10870 At1g18640 |
| nitrogen compound biosynthetic process | 8.5 | 1 | 0.067 | At5g16290 At5g66120 At5g10870 At1g18640 |
| cellular process | 59.6 | 36.8 | 0.071 | At3g05230 At4g33250 At4g04770 At2g22570 At1g64880 At3g46560 At1g18640 At5g06910 At3g44680 At5g16290 At5g66120 At1g09760 At5g10870 At4g24920 At4g25080 At2g45200 At3g18380 At1g51510 At5g03455 At1g56450 At1g11860 At3g17100 At4g37280 At3g52730 At4g25280 At3g10670 At1g32100 At1g18800 |
| metabolism of the cysteine - aromatic group | 8.5 | 0.8 | 0.032 | At1g18640 At5g10870 At1g11860 At5g66120 |
| Node 51 |  |  |  |  |
| lipid transport | 4.4 | 0.3 | 0.049 | At3g27870 At3g22600 At1g31812 |
| Node 52 |  |  |  |  |
| detoxification | 2.6 | 1 | 0.027 | At2g31570 At1g10360 At5g18600 At5g39950 At4g33040 At1g19570 At3g17880 At3g08710 At4g31870 At1g07960 At1g76080 At1g63460 At5g62480 At5g13810 At3g52960 At1g04980 At5g03455 |
| oxygen and radical detoxification | 2.5 | 0.9 | 0.046 | At1g76080 At2g31570 At1g10360 At5g18600 At5g62480 At1g63460 At5g39950 At4g33040 At1g19570 At3g17880 At3g08710 At5g13810 At3g52960 At4g31870 At1g04980 At1g07960 |
| Node 53 |  |  |  |  |
| cellular biosynthetic process | 10.1 | 6.8 | 0.001 | At3g62120 At4g14690 At4g13780 At5g23535 At1g07320 At3g63190 At1g64880 At2g43030 At5g13510 At1g01480 At1g75350 At3g47930 At1g35680 At4g11120 At1g30120 At3g18880 At5g45930 At5g35790 At5g13450 At3g54470 At3g25920 At4g01690 At1g70190 At5g26030 At5g67030 At3g60880 At5g18110 At2g44860 At2g44620 At2g39770 At4g22260 At1g17745 At2g48120 At2g21790 At1g71350 At5g61670 At5g25780 At2g40290 At5g54600 At5g53070 At1g52300 At4g01900 At5g10870 At1g15710 At5g11750 At2g20690 At5g58240 At5g06360 At3g24200 At5g61170 At3g63250 At1g05190 At5g46160 At3g54210 At5g16230 At4g33250 At2g46580 At5g64650 At1g80750 At4g20930 At1g70570 At5g54940 At5g38830 At1g70600 At2g29690 At4g39880 At5g03730 At4g34850 At1g60440 At3g56940 At1g63660 At2g38670 At1g03475 At5g08170 At1g09795 At1g16350 At4g04320 At4g02610 At3g63410 At3g27850 At4g31180 At1g32100 At5g14800 At3g14990 At2g43360 At1g62360 At3g06040 At1g31860 At3g51770 At2g18250 At3g51820 At5g66860 At1g22940 At2g27490 At3g63490 At1g06380 At2g22230 At1g64190 At5g30510 At5g40950 At1g55670 At1g25260 At2g20060 At4g14210 At5g67510 At3g13120 |
| translation | 4.5 | 2.7 | 0.041 | At3g62120 At4g33250 At4g13780 At5g23535 At1g07320 At5g64650 At1g80750 At3g63190 At1g64880 At2g43030 At1g70600 At5g38830 At5g54940 At4g39880 At5g13510 At1g75350 At1g35680 At4g11120 At3g18880 At3g25920 At4g31180 At3g27850 At1g70190 At5g18110 At2g44860 At3g06040 At1g71350 At5g25780 At2g40290 At5g54600 At5g53070 At5g66860 At1g52300 At5g11750 At3g63490 At1g06380 At5g06360 At5g61170 At5g30510 At5g40950 At1g25260 At5g46160 At1g05190 At2g20060 At3g13120 At3g54210 At5g67510 |
| ribosome biogenesis | 3.6 | 1.5 | 0 | At1g52300 At2g43030 At5g64650 At1g70600 At1g35680 At5g23535 At1g70190 At5g13510 At5g61170 At5g46160 At3g06040 At5g53070 At1g25260 At3g18880 At5g55140 At5g30510 At1g07320 At3g22660 At3g57000 At5g40950 At5g06360 At5g20180 At1g64880 At1g80750 At3g25920 At5g11750 At3g13120 At2g20060 At1g75350 At3g54210 At5g67510 At1g05190 At4g39880 At2g44860 At1g32990 At5g54600 At3g27850 At3g63490 |
| translation | 4.1 | 2 | 0 | At1g52300 At1g71350 At2g43030 At5g64650 At5g54940 At1g70600 At1g35680 At4g33250 At5g23535 At1g70190 At5g13510 At5g61170 At5g46160 At3g06040 At5g53070 At1g25260 At3g18880 At5g30510 At1g07320 At5g40950 At5g06360 At1g64880 At1g80750 At5g11750 At3g13120 At3g25920 At2g20060 At1g20575 At1g75350 At5g18110 At4g31180 At5g25780 At4g11120 At3g54210 At2g23350 At5g67510 At1g05190 At4g39880 At2g44860 At3g63190 At5g54600 At3g27850 At3g63490 |
| protein synthesis | 5 | 2.8 | 0.001 | At1g52300 At1g71350 At2g43030 At3g62120 At2g40660 At5g64650 At5g54940 At1g70600 At1g35680 At4g33250 At5g23535 At1g70190 At5g13510 At5g38830 At5g61170 At5g46160 At3g06040 At5g53070 At1g25260 At3g18880 At5g55140 At5g30510 At1g18950 At1g07320 At3g22660 At3g57000 At5g40950 At5g06360 At5g20180 At1g64880 At1g80750 At3g13120 At5g11750 At3g25920 At2g20060 At1g20575 At1g75350 At5g18110 At4g31180 At4g13780 At5g25780 At4g11120 At3g54210 At2g23350 At5g67510 At1g05190 At4g39880 At2g44860 At3g63190 At5g54600 At1g32990 At3g27850 At3g63490 |
| Node 54 |  |  |  |  |
| mitochondrial transport | 1.4 | 0.3 | 0.08 | At3g49560 At4g16160 At2g46470 At5g50810 At3g25120 At3g20000 At1g20350 |
| translation | 4.3 | 2 | 0.098 | At5g18110 At5g41520 At3g07300 At4g33250 At1g35680 At3g57190 At3g53890 At5g61170 At5g46160 At3g57290 At4g11120 At1g15390 At5g30510 At1g05190 At5g40950 At3g01790 At5g06360 At1g64880 At5g11750 At3g25920 At2g39820 At5g08180 |
| Node 55 |  |  |  |  |
| electron transport | 7.3 | 2.7 | 0.074 | At1g10960 At5g03630 At5g17170 At1g20340 At5g20080 At2g24940 At5g67590 At3g08710 At1g77370 At3g54660 At1g65840 At4g22260 At5g37510 At4g27500 |
| Node 56 |  |  |  |  |
| mitochondrial transport | 8 | 0.3 | 0.072 | At4g39850 At3g20000 |
| lipid/fatty acid transport | 8 | 0.3 | 0.081 | At2g44620 At4g39850 |
| Node 57 |  |  |  |  |
| apoptosis | 3.8 | 0.1 | 0.083 | At3g07190 At5g47120 |
| Node 59 |  |  |  |  |
| cellular biosynthetic process | 13.6 | 6.8 | 0.004 | At4g33250 At2g04520 At1g64880 At1g48605 At2g43030 At3g57190 At5g17230 At5g66120 At3g02360 At4g30690 At1g78630 At5g08170 At3g54470 At3g63410 At4g31180 At5g67030 At2g43360 At1g48350 At2g44620 At1g50480 At1g01610 At1g18640 At5g49030 At5g10870 At3g14110 At1g15710 At3g08740 At5g11750 At3g63490 At5g61170 At2g22230 At5g40950 At5g46160 At1g15390 At2g19720 At3g54210 At1g65290 At2g20060 |
| translation | 7.1 | 2.7 | 0.008 | At4g33250 At1g48350 At2g04520 At1g64880 At2g43030 At3g57190 At5g49030 At3g08740 At5g11750 At1g78630 At4g30690 At3g63490 At5g61170 At5g40950 At4g31180 At1g15390 At5g46160 At2g19720 At3g54210 At2g20060 |
| cellular protein metabolic process | 16.1 | 9.7 | 0.072 | At4g33250 At2g04520 At3g60210 At1g30950 At2g16920 At1g64880 At1g73660 At2g43030 At5g60750 At1g12410 At3g57190 At1g17280 At1g06900 At3g01050 At3g60820 At4g30690 At1g78630 At1g67890 At5g03455 At5g22130 At2g07340 At4g31180 At5g60160 At2g35360 At1g02560 At1g48350 At3g05230 At1g29990 At2g39960 At1g63770 At5g49030 At1g52600 At5g11750 At3g08740 At1g32750 At3g63490 At5g61170 At1g56450 At5g40950 At1g15390 At5g46160 At2g19720 At3g08980 At3g54210 At2g20060 |
| protein metabolic process | 16.4 | 10.1 | 0.088 | At4g33250 At2g04520 At3g60210 At1g30950 At2g16920 At1g64880 At1g73660 At2g43030 At5g60750 At1g12410 At3g57190 At1g17280 At1g06900 At3g01050 At3g60820 At4g30690 At1g78630 At1g67890 At5g03455 At5g22130 At2g07340 At4g31180 At5g60160 At2g35360 At1g02560 At1g48350 At3g05230 At5g55630 At1g29990 At2g39960 At1g63770 At5g49030 At1g52600 At5g11750 At3g08740 At1g32750 At3g63490 At5g61170 At1g56450 At5g40950 At5g46160 At1g15390 At2g19720 At2g20060 At3g08980 At3g54210 |
| translation | 7.1 | 2 | 0 | At2g43030 At4g33250 At3g57190 At2g04520 At5g61170 At5g46160 At4g31180 At2g19720 At1g48350 At3g54210 At1g15390 At4g30690 At5g40950 At1g64880 At5g11750 At3g63490 At1g78630 At3g08740 At5g08180 At2g20060 |
| cytoplasm | 10 | 4 | 0.001 | At3g01150 At5g47120 At2g43030 At3g60820 At1g80050 At4g33250 At1g30950 At5g61170 At5g46160 At4g31180 At1g18800 At4g18930 At4g09010 At2g19720 At1g48350 At1g31812 At3g54210 At1g48410 At5g10870 At5g40950 At1g56450 At1g64880 At1g76990 At5g11750 At1g78630 At3g63490 At1g31910 At5g08180 |
| protein synthesis | 7.5 | 2.8 | 0.004 | At2g43030 At4g33250 At3g57190 At2g04520 At5g61170 At5g46160 At4g31180 At2g19720 At1g48350 At3g54210 At5g49030 At1g15390 At4g30690 At5g40950 At1g64880 At5g11750 At3g63490 At1g78630 At3g08740 At5g08180 At2g20060 |
| ribosome biogenesis | 4.6 | 1.5 | 0.033 | At2g43030 At5g40950 At5g61170 At5g46160 At1g64880 At2g19720 At5g11750 At1g78630 At3g63490 At5g08180 At1g48350 At2g20060 At3g54210 |
| Node 60 |  |  |  |  |
| fatty acid metabolism | 1.8 | 0.6 | 0.087 | At2g38040 At5g48880 At5g16390 At4g13050 At1g36050 At1g65290 At1g30120 At2g44620 At3g15870 At1g04290 At1g01710 At3g61200 |
| Node 61 |  |  |  |  |
| aromatic compound biosynthetic process | 4.9 | 0.5 | 0.013 | At1g07780 At4g02610 At5g66120 At3g26900 At1g32100 |
| aromatic amino acid family biosynthetic process | 3.9 | 0.3 | 0.014 | At1g07780 At4g02610 At5g66120 At3g26900 |
| chorismate metabolic process | 3.9 | 0.3 | 0.014 | At1g07780 At4g02610 At5g66120 At3g26900 |
| amino acid biosynthetic process | 5.8 | 0.8 | 0.015 | At1g07780 At4g02610 At5g16290 At5g66120 At3g26900 At1g18640 |
| aromatic amino acid family metabolic process | 3.9 | 0.3 | 0.018 | At1g07780 At4g02610 At5g66120 At3g26900 |
| amine biosynthetic process | 5.8 | 0.9 | 0.023 | At1g07780 At4g02610 At5g16290 At5g66120 At3g26900 At1g18640 |
| nitrogen compound biosynthetic process | 5.8 | 1 | 0.039 | At1g07780 At4g02610 At5g16290 At5g66120 At3g26900 At1g18640 |
| dicarboxylic acid metabolic process | 3.9 | 0.4 | 0.042 | At1g07780 At4g02610 At5g66120 At3g26900 |
| hyperosmotic response | 2.9 | 0.2 | 0.054 | At5g19660 At2g40950 At1g48605 |
| hyperosmotic salinity response | 2.9 | 0.2 | 0.054 | At5g19660 At2g40950 At1g48605 |
| aromatic compound metabolic process | 5.8 | 1.1 | 0.069 | At1g07780 At4g02610 At5g66120 At3g26900 At1g32100 At1g50480 |
| metabolism of tryptophan | 3.9 | 0.3 | 0.02 | At3g26900 At1g07780 At5g66120 At4g02610 |
| biosynthesis of tryptophan | 2.9 | 0.2 | 0.043 | At1g07780 At5g66120 At4g02610 |
| Node 63 |  |  |  |  |
| metabolism of threonine | 1.1 | 0.1 | 0.07 | At5g21060 At3g10050 At1g14810 |
| Node 64 |  |  |  |  |
| metabolism of threonine | 1 | 0.1 | 0.098 | At5g21060 At3g10050 At1g14810 |
| Node 65 |  |  |  |  |
| sulfur compound catabolic process | 4.9 | 0 | 0.002 | At4g29210 At1g64660 |
| photosynthesis | 4.9 | 0.2 | 0.085 | At4g04020 At4g14690 |
| Node 76 |  |  |  |  |
| secondary metabolism | 22.2 | 1.4 | 0.071 | At2g04850 At3g03190 |
| Node 77 |  |  |  |  |
| ARF protein signal transduction | 3.8 | 0.1 | 0.084 | At5g19610 At3g07940 |
| &regulation of ARF protein signal transduction | 3.8 | 0.1 | 0.084 | At5g19610 At3g07940 |
| oxidative stress response | 7.5 | 0.5 | 0.007 | At4g02580 At3g03190 At2g46370 At4g00860 |
| Node 88 |  |  |  |  |
| protein/peptide degradation | 5 | 3 | 0.083 | At5g16310 At5g40560 At1g66670 At1g51550 At2g34940 At1g02170 At1g08750 At1g17110 At4g30640 At3g62940 At3g59950 At3g02875 At3g47890 At1g52600 At5g51740 At4g30890 At5g04200 At3g02070 At1g12410 At5g26860 At2g26990 At1g76920 At1g24140 At1g67420 At5g20660 At2g25740 At4g20850 At5g60160 At5g15250 At5g54140 At2g16920 At5g35220 At5g43600 At5g03330 At5g46390 At3g06330 At1g02560 At1g55860 At4g37040 At5g64240 At1g35340 |
| Node 90 |  |  |  |  |
| glutathione conjugation reaction | 2.6 | 0.2 | 0.087 | At3g54660 At2g02380 At3g03190 |
| Node 93 |  |  |  |  |
| cellular process | 50.7 | 36.8 | 0.002 | At3g01170 At1g48410 At1g06950 At1g27700 At2g05990 At3g46100 At3g60210 At2g43030 At3g16110 At3g15390 At3g47930 At2g26990 At5g35520 At3g59020 At1g10610 At2g47390 At5g08030 At2g03870 At4g14800 At3g26720 At5g08180 At5g16560 At1g03330 At5g20180 At2g01750 At1g01520 At3g22480 At1g48870 At4g01690 At4g13050 At3g03610 At5g18110 At5g23420 At1g09130 At4g16800 At2g38140 At2g44620 At1g21630 At1g72550 At4g17040 At1g59820 At5g10480 At3g53890 At5g54140 At1g12370 At5g61670 At2g39960 At5g05080 At2g28100 At5g57780 At1g76310 At5g10870 At4g27490 At1g25220 At4g05440 At5g08160 At5g58520 At2g44950 At5g62600 At4g28880 At4g15760 At1g09020 At5g16230 At4g33510 At2g34720 At2g20860 At4g04020 At5g39785 At3g52280 At4g31540 At1g63650 At5g47370 At5g15250 At5g54200 At1g07770 At1g08980 At2g25940 At5g23600 At4g17710 At1g51510 At2g33630 At1g32990 At1g03000 At5g22080 At5g60160 At1g06070 At2g26230 At1g32100 At3g03190 At2g05810 At1g02560 At2g39630 At3g61740 At2g18290 At1g76550 At4g04940 At4g20410 At4g18390 At2g41500 At2g24120 At3g01820 At1g65700 At1g17110 At4g15417 At2g45200 At2g38040 At3g63490 At2g24500 At2g22230 At5g05920 At1g42550 At1g69580 At4g14210 |
| cellular metabolic process | 40.8 | 29.1 | 0.013 | At3g01170 At1g48410 At2g05990 At3g46100 At3g60210 At2g43030 At3g15390 At3g47930 At2g26990 At3g59020 At1g10610 At2g47390 At5g08030 At2g03870 At4g14800 At3g26720 At1g03330 At1g01520 At3g22480 At4g01690 At4g13050 At5g18110 At5g23420 At1g09130 At4g16800 At2g38140 At2g44620 At1g21630 At1g72550 At4g17040 At5g10480 At3g53890 At5g54140 At1g12370 At5g61670 At2g39960 At5g05080 At2g28100 At5g57780 At5g10870 At4g27490 At1g25220 At5g08160 At5g58520 At2g44950 At5g62600 At4g28880 At4g15760 At5g16230 At2g34720 At2g20860 At4g33510 At4g04020 At5g39785 At1g63650 At5g47370 At5g15250 At1g07770 At1g08980 At2g25940 At5g23600 At4g17710 At1g51510 At2g33630 At1g03000 At5g22080 At5g60160 At1g06070 At2g26230 At1g32100 At3g03190 At2g05810 At1g02560 At2g39630 At3g61740 At1g76550 At4g04940 At4g18390 At2g41500 At2g24120 At3g01820 At1g65700 At1g17110 At4g15417 At3g63490 At2g38040 At2g22230 At2g24500 At5g05920 At1g69580 At4g14210 |
| primary metabolic process | 38.6 | 27.5 | 0.024 | At3g01170 At2g05990 At3g46100 At3g60210 At2g43030 At3g15390 At2g26990 At3g59020 At1g10610 At2g47390 At2g03870 At4g14800 At3g26720 At1g03330 At1g01520 At3g22480 At4g13050 At5g18110 At5g23420 At1g09130 At4g16800 At2g38140 At2g44620 At1g21630 At4g17040 At1g72550 At3g11210 At4g25370 At5g10480 At3g53890 At5g54140 At1g12370 At5g61670 At2g39960 At5g05080 At5g57780 At5g10870 At4g27490 At1g25220 At5g08160 At5g58520 At2g44950 At5g62600 At4g28880 At3g58790 At5g16230 At2g34720 At2g20860 At4g33510 At1g03520 At5g39785 At1g63650 At5g47370 At5g15250 At1g07770 At2g25940 At5g23600 At4g17710 At1g51510 At2g33630 At1g03000 At5g22080 At5g60160 At1g06070 At2g26230 At1g32100 At2g05810 At1g02560 At2g39630 At3g61740 At1g76550 At4g04940 At4g18390 At2g41500 At2g24120 At3g01820 At1g65700 At1g17110 At4g15417 At3g63490 At2g38040 At2g22230 At2g24500 At5g05920 At1g69580 At4g14210 |
| auxin metabolic process | 1.8 | 0.1 | 0.025 | At1g48410 At5g54140 At1g08980 At1g25220 |
| organic acid biosynthetic process | 3.1 | 0.6 | 0.029 | At2g38040 At2g05990 At5g16230 At4g33510 At2g22230 At2g44620 At4g13050 |
| carboxylic acid biosynthetic process | 3.1 | 0.6 | 0.029 | At2g38040 At2g05990 At5g16230 At4g33510 At2g22230 At2g44620 At4g13050 |
| metabolic process | 45.7 | 34.4 | 0.036 | At3g01170 At1g48410 At2g05990 At5g11340 At3g46100 At3g60210 At2g43030 At3g15390 At3g47930 At3g27870 At2g26990 At2g41040 At3g59020 At1g10610 At2g47390 At5g08030 At2g03870 At4g14800 At3g26720 At1g03330 At1g01520 At3g22480 At4g01690 At4g13050 At5g18110 At5g23420 At1g09130 At4g16800 At2g38140 At2g44620 At1g21630 At1g72550 At4g17040 At3g11210 At4g25370 At5g10480 At3g53890 At5g54140 At1g12370 At5g61670 At2g39960 At5g05080 At2g28100 At5g57780 At5g10870 At4g27490 At1g25220 At5g08160 At5g58520 At2g44950 At5g62600 At4g28880 At4g15760 At3g58790 At5g16230 At4g33510 At2g34720 At2g20860 At1g03520 At4g04020 At5g39785 At1g63650 At1g01790 At5g47370 At3g17365 At5g15250 At1g07770 At1g08980 At2g25940 At5g23600 At4g17710 At1g51510 At5g04930 At2g33630 At1g03000 At5g22080 At5g60160 At1g06070 At2g26230 At1g32100 At3g03190 At2g05810 At1g02560 At2g39630 At3g61740 At1g76550 At4g04940 At4g18390 At2g41500 At2g24120 At3g01820 At3g09810 At1g65700 At1g17110 At4g15417 At3g63490 At2g38040 At2g24500 At2g22230 At5g05920 At1g69580 At4g14210 |
| cell fate commitment | 1.3 | 0.1 | 0.04 | At5g16560 At1g63650 At2g41500 |
| fatty acid metabolic process | 3.6 | 0.8 | 0.042 | At2g38040 At2g05990 At5g16230 At4g16800 At1g03000 At2g22230 At2g44620 At4g13050 |
| fatty acid biosynthetic process | 2.7 | 0.4 | 0.046 | At2g38040 At2g05990 At5g16230 At2g22230 At2g44620 At4g13050 |
| fatty acid binding (e.g. acyl-carrier protein) | 1.3 | 0.1 | 0.009 | At4g24230 At1g31812 At2g05990 |
| fatty acid metabolism | 3.1 | 0.6 | 0.052 | At2g38040 At2g44620 At2g22230 At4g13050 At2g05990 At5g16230 At4g16800 |
| protein processing (proteolytic) | 2.2 | 0.3 | 0.075 | At1g09130 At1g02560 At2g39960 At2g25940 At4g17040 |
| Node 94 |  |  |  |  |
| cell fate commitment | 1.1 | 0.1 | 0.078 | At5g16560 At1g63650 At2g41500 |
| cellular process | 46.5 | 36.8 | 0.079 | At1g27700 At2g05990 At3g01170 At1g48410 At1g06950 At3g07690 At3g09880 At3g46100 At3g60210 At2g43030 At3g16110 At3g58600 At3g15390 At3g47930 At5g35520 At2g26990 At3g28970 At3g59020 At5g08030 At1g10610 At2g47390 At2g03870 At4g14800 At3g26720 At5g08180 At5g20180 At5g16560 At1g03330 At2g01750 At3g22480 At4g20070 At1g48870 At4g01690 At3g03610 At4g13050 At5g18110 At5g23420 At1g09130 At4g16800 At2g38140 At3g16630 At2g44620 At1g72550 At1g21630 At1g59820 At4g15520 At5g10480 At3g53890 At5g54140 At1g12370 At5g61670 At2g39960 At4g37190 At2g28100 At5g05080 At5g57780 At1g76310 At5g10870 At4g27490 At1g25220 At5g08160 At4g05440 At5g08200 At5g58520 At2g44950 At5g49230 At4g28880 At5g62600 At4g15760 At1g09020 At5g16230 At4g33510 At2g20860 At2g34720 At1g30950 At5g63910 At4g04020 At5g39785 At3g52280 At1g63650 At4g31540 At5g47370 At4g04880 At5g15250 At5g54200 At1g07770 At2g25940 At4g17710 At5g23600 At2g33630 At1g51510 At1g32990 At1g03000 At1g09795 At1g77470 At5g22080 At5g60160 At2g26230 At1g06070 At1g32100 At3g03190 At2g05810 At1g02560 At2g39630 At2g18290 At3g61740 At1g76550 At4g04940 At4g04610 At4g20410 At4g18390 At3g01820 At2g24120 At2g41500 At1g65700 At1g17110 At4g15417 At2g45200 At2g38040 At3g63490 At2g22230 At2g24500 At5g40950 At5g05920 At1g42550 At1g69580 At4g14210 |
| Node 95 |  |  |  |  |
| cellular process | 45.8 | 36.8 | 0.085 | At3g10220 At3g54860 At1g07780 At2g28560 At3g43980 At2g40730 At1g68640 At4g02070 At2g34940 At3g03610 At4g13050 At3g13940 At3g16630 At1g06200 At5g15910 At3g57610 At4g00240 At5g45680 At5g10480 At5g08550 At5g61670 At1g72970 At5g22030 At2g37970 At1g09300 At4g01900 At3g01180 At4g20380 At2g16440 At5g08160 At1g66430 At5g09420 At5g26240 At5g49230 At1g36280 At4g33250 At2g04520 At5g25480 At4g04020 At5g39785 At5g63960 At1g32200 At5g24260 At1g63650 At3g20000 At3g25990 At4g24350 At5g48630 At1g09795 At4g38020 At4g17760 At2g01720 At5g23090 At3g63410 At2g07340 At3g10050 At5g17410 At4g27940 At5g22800 At3g61130 At4g33200 At4g04610 At5g10240 At5g22370 At4g36040 At1g11000 At1g09760 At4g22200 At4g30580 At1g42550 At3g07160 At3g13120 At1g06950 At1g15220 At1g06720 At3g06580 At5g64860 At2g30710 At1g14810 At1g80920 At3g13210 At1g55860 At5g13570 At3g18630 At3g47930 At2g46090 At2g42120 At1g48635 At5g08030 At2g18620 At2g40490 At1g31180 At5g19610 At3g17940 At1g05820 At5g62410 At4g11130 At3g11240 At3g24350 At5g04130 At2g39450 At5g65830 At1g69220 At3g59890 At4g15520 At1g72470 At1g28090 At1g12370 At5g25780 At3g18165 At2g31650 At5g14250 At4g21070 At2g37500 At3g61960 At2g06210 At1g51160 At1g77930 At1g51940 At1g17040 At4g33060 At1g31190 At2g03720 At5g59610 At3g57660 At2g37090 At5g51700 At1g26840 At3g04820 At2g02380 At3g47610 At5g16750 At3g46920 At3g61530 At5g60160 At2g47420 At4g02580 At5g14800 At5g49970 At4g23100 At3g22200 At4g29010 At5g16310 At2g45200 At5g14620 At5g48600 At3g24440 At3g02590 At1g79850 At1g25260 At1g18800 |
| Node 98 |  |  |  |  |
| translation elongation | 2.2 | 0.1 | 0.048 | At4g11120 At5g39900 At1g20575 |
| Node 99 |  |  |  |  |
| unannotated | 37 | 14.2 | 0.024 | At5g08740 At1g35860 At1g59560 At3g06810 At1g13820 At3g54600 At5g15330 At3g62860 At1g50410 At5g61140 |
| aromate metabolism | 7.4 | 0.3 | 0.08 | At3g62860 At1g13820 |
