# Supplementary material for: A Functional Phylogenomic View of the Seed Plants
Source: PLoS Genet. 2011 Dec 15;7(12):e1002411. doi: 10.1371/journal.pgen.1002411 (PMC3240601; doi:10.1371/journal.pgen.1002411)
Supplement: Table S4 — Log-likelihood scores for inferences based on the CAT and GAMMA rate heterogeneity models from different starting trees under the GTR substitution matrix. Inferences that yielded better likelihood scores in CAT-GAMMA comparisons are shaded for clarity. (DOC) [file pgen.1002411.s011.doc]

**Table S4. Log-likelihood scores for inferences based on the CAT and GAMMA among-site rate heterogeneity models from different starting trees under the GTR substitution matrix.** Inferences that yielded better likelihood scores in CAT-GAMMA comparisons are shaded for clarity.

| **MP starting trees** | | **Random starting trees** | |
| --- | --- | --- | --- |
| CAT | GAMMA | CAT | GAMMA |
| -35802926.130201 | -35802973.588424 | -35803089.638393 | -35804253.076881 |
| -35802927.922231 | -35802929.909993 | -35803652.117774 | -35803555.762021 |
| -35802923.520383 | -35803940.961466 | -35803709.872131 | -35804048.176038 |
| -35802950.556227 | -35802969.534089 | -35803624.985630 | -35803587.622515 |
| -35802925.236462 | -35802936.691470 | -35809763.018741 | -35803518.136397 |
