# Supplementary material for: A Functional Phylogenomic View of the Seed Plants
Source: PLoS Genet. 2011 Dec 15;7(12):e1002411. doi: 10.1371/journal.pgen.1002411 (PMC3240601; doi:10.1371/journal.pgen.1002411)
Supplement: Table S6 — Selection analysis results for the Euphorbia candidate genes. Values given for 13 genes whose amino acid sequence showed strong, positive PBS (PBS>10) and belonged to the same MIPS term. (DOC) [file pgen.1002411.s013.doc]

**Table S6. Selection analysis results for the *Euphorbia* candidate genes.** Values given for 13 genes whose amino acid sequence showed strong, positive PBS (PBS>10) and belonged to the same MIPS term.

| **Genes*** | ***dN*/*dS*** | | | | | | **No. of codons with significant *dN* change along the branch leading to *Euphorbia*** |  | | ***dS*** | |  |
| --- | --- | --- | --- | --- | --- | --- | --- | --- | --- | --- | --- | --- |
|  | **gene-wide** | **mean** | | **median** | | **skewness** |  | **mean** | | **median** | | **skewness** |
| At1g65280 | 0.361 | 9.675 | 0.262 | | 6.331 | | 4 | 167.859 | 2.326 | | 7.351 | |
| At3g54960 | 0.219 | 0.464 | 0.133 | | 5.423 | | 5 | 500.680 | 2.813 | | 4.241 | |
| At4g31870 | 0.377 | 0.513 | 0.240 | | 5.777 | | 7 | 252.386 | 2.378 | | 6.223 | |
| At2g31570 | 0.236 | 48.901 | 0.192 | | 15.297 | | 6 | 128.219 | 2.708 | | 8.978 | |
| At1g20620 | 0.138 | 0.334 | 0.018 | | 16.989 | | 21 | 440.738 | 3.804 | | 4.632 | |
| At5g23310 | 0.389 | 0.601 | 0.266 | | 13.280 | | 14 | 238.031 | 2.697 | | 6.391 | |
| At3g15360 | 0.410 | 0.734 | 0.341 | | 4.985 | | 8 | 168.623 | 2.247 | | 8.058 | |
| At1g76080 | 0.336 | 1.700 | 0.189 | | 19.168 | | 24 | 169.028 | 2.850 | | 7.808 | |
| At2g47730 | 0.276 | 0.526 | 0.209 | | 5.701 | | 13 | 104.529 | 2.238 | | 9.994 | |
| At4g33040 | 0.349 | 0.621 | 0.216 | | 4.320 | | 3 | 685.118 | 2.658 | | 3.311 | |
| At1g64500 | 0.365 | 32.155 | 0.183 | | 14.438 | | 25 | 621.311 | 2.103 | | 3.674 | |
| At1g19570 | 0.269 | 9.211 | 0.299 | | 18.067 | | 4 | 155.420 | 2.307 | | 8.151 | |
| At3g27820 | 0.209 | 0.431 | 0.128 | | 13.394 | | 18 | 195.490 | 3.262 | | 7.124 | |

* *Arabidopsis* gene symbols
