# Supplementary material for: A Functional Phylogenomic View of the Seed Plants
Source: PLoS Genet. 2011 Dec 15;7(12):e1002411. doi: 10.1371/journal.pgen.1002411 (PMC3240601; doi:10.1371/journal.pgen.1002411)
Supplement: Text S1 — Supporting Methods. (PDF) [file pgen.1002411.s014.pdf]

SUPPORTING INFORMATION

Text S1. Supporting Methods

Phylogenetic analysis – Maximum Likelihood (ML). In order to determine the best-scoring ML protein substitution model we optimized ML model parameters (branch lengths and alpha shape parameter of the Γ-distribution model of among-site rate heterogeneity) on the shortest maximum parsimony (MP) tree using the Pthreads-based development version of RAxML for all 11 protein models implemented in the program, including the recent LG model [1] and the respective variants using empirical amino acid residue frequencies (F). This new version of RAxML was specifically adapted to the computational requirements of this analysis and can handle datasets exceeding a 100 GB memory footprint – our dataset requires 89.2 GB of main memory under the GAMMA (Γ) model of among-site rate heterogeneity.

Moreover, we conducted an ML estimate of a dedicated amino acid General Time-Reversible (GTR) substitution matrix, GreenREV (Table S5), to infer the 189 rate parameters on the shortest MP tree using the Pthreads-based version of RAxML. Note that, due to the size of the input dataset, unlike with smaller protein alignments, it is highly unlikely to overparameterize the method by deriving a likelihood-based GTR matrix. The dedicated GTR substitution model yielded significantly better likelihood scores than the best standard model (JTT with empirical amino acid frequencies [F]) – the

latter model being chosen after it scored better than the remaining 21 models available in RAxML.

Next, we conducted a total of 20 ML searches for the best-scoring ML tree under the CAT approximation of rate heterogeneity and the GAMMA model of rate heterogeneity using the most recent RAxML hill-climbing algorithm [2]. For both models we used the same 10 random starting trees and 10 randomized stepwise addition MP starting trees in order to determine the impact of CAT and GAMMA onto the final scores, that were computed under GAMMA. We found that inferences under CAT always yielded slightly better GAMMA-based likelihood scores than inferences under GAMMA on MP starting trees, while for random starting trees CAT is better in 2 out of 5 trees (Table S4).

We further evaluated the effect of starting tree topology by using the best MP tree found with PAUP*. Log-likelihood scores were better in both CAT and GAMMA models ($logLik_{CAT}$ = –35,802,561.535669, $logLik_{GAMMA}$ = –35,802,415.779498) with the latter model yielding the best likelihood scores across all inferences.

Since CAT returns better scores on MP starting trees which are also used as starting trees for bootstrap replicates, and due to the significantly lower resource requirements of CAT (memory footprint = 22 GB) and approximately 3 times shorter inference times (e.g. ~12 hours on 128 cores for CAT, ~40 hours on 128 cores for GAMMA on an MP starting tree), we therefore used CAT for all subsequent bootstrap searches. While it has been suggested that more than 4 discrete GAMMA rate categories should be used for analyses

of large and complex datasets, this is not feasible because the memory requirements and inference times for the GAMMA model grow linearly with the number of discrete rates, i.e. a single phylogenetic tree search with 8 discrete rates would have required approximately 178.4 GB memory and 80 hours on 128 cores. Due to the still limited computational resources at our disposal, we executed the novel rapid RAxML bootstrap algorithm to compute 223 non-parametric rapid bootstrap (RBS) pseudo-replicates. In order to determine if we conducted a sufficient number of RBS replicates we applied our novel bootstrap convergence test [3,4] implemented in RAxML to our collection of RBS trees. Pattengale et al. [3,4] devised the bipartition Frequency Criterion (FC) that conducts a permutation test on 100 random splits of the bootstrap replicates into equal halves and computes the Pearson correlation coefficient on all bipartitions induced by the respective splits. According to the FC criterion the bootstrap search converged after 122 replicates. In addition to the FC, we applied Pattengale et al.'s [3,4] Weighted Criterion (WC) that conducts a permutation test on 100 random splits of the bootstrap replicates into equal halves and computes the Weighted Robinson-Foulds (WRF) distance [5] between the extended majority rule consensus trees induced by the respective splits. We used a threshold setting of 2%, i.e. at the point of convergence the WRF distance between consensus trees for 99 out of 100 splits must be less or equal to 2%. The WC converged after 74 replicates. Note that the test can only be applied to even numbers of replicates, since our permutation test requires a split of bootstrap trees into halves of equal size.

Empirical amino acid frequencies of the GreenREV matrix:

A=0.068923, R=0.054652, N=0.042172, D=0.052019

C=0.025410, Q=0.039735, E=0.060227, G=0.063082

H=0.030202, I=0.051412, L=0.084339, K=0.057721

M=0.029435, F=0.043987, P=0.051281, S=0.077156

T=0.049716, W=0.021853, Y=0.033453, V=0.063224

References

1. Le SQ, Gascuel O (2008) An improved general amino acid replacement matrix. Mol Biol Evol 25: 1307-1320.

2. Stamatakis A, Blagojevic F, Nikolopoulos DS, Antonopoulos CD (2007) Exploring new search algorithms and hardware for phylogenetics: RAxML meets the IBM Cell. J VLSI Signal Process 48: 271-286.

3. Pattengale N, Alipour M, Bininda-Emonds O, Moret B, Stamatakis A (2009) How many bootstrap replicates are necessary? Research in Computational Molecular Biology (RECOMB). Tucson, AZ. pp. 184-200.

4. Pattengale ND, Alipour M, Bininda-Emonds ORP, Moret BME, Stamatakis A (2010) How many bootstrap replicates are necessary? J Comput Biol 17: 337-354.

5. Robinson D, Foulds L (1979) Comparison of weighted labelled trees. In: Dold A, Eckmann B, editors. Combinatorial Mathematics VI. Heidelberg: Springer-Verlag. pp. 119-126.
